# Factors associated with psychiatric and physical comorbidities in bipolar disorder: a nationwide multicenter cross-sectional observational study

**DOI:** 10.3389/fpsyt.2023.1208551

**Published:** 2023-07-25

**Authors:** Jozef Dragasek, Michal Minar, Peter Valkovic, Maria Pallayova

**Affiliations:** ^1^1st Department of Psychiatry, University Hospital of Louis Pasteur and Pavol Jozef Safarik University Faculty of Medicine, Kosice, Slovakia; ^2^2nd Department of Neurology, Faculty of Medicine, Comenius University in Bratislava, University Hospital Bratislava, Bratislava, Slovakia; ^3^Centre of Experimental Medicine, Institute of Normal and Pathological Physiology, Slovak Academy of Sciences, Bratislava, Slovakia; ^4^Department of Human Physiology, Faculty of Medicine, Pavol Jozef Safarik University, Kosice, Slovakia

**Keywords:** bipolar disorder, adult, age of onset, comorbidity, prevalence, antipsychotics, olanzapine, phenotype

## Abstract

**Background:**

Bipolar disorder (BD) is a chronic and disabling affective disorder with significant morbidity and mortality. Despite the high rate of psychiatric and physical health comorbidity, little is known about the complex interrelationships between clinical features of bipolar illness and comorbid conditions. The present study sought to examine, quantify and characterize the cross-sectional associations of psychiatric and physical comorbidities with selected demographic and clinical characteristics of adults with BD.

**Methods:**

A nationwide multicenter cross-sectional observational epidemiological study conducted from October 2015 to March 2017 in Slovakia.

**Results:**

Out of 179 study participants [median age 49 years (interquartile range IQR 38–58); 57.5% females], 22.4% were free of comorbidity, 42.5% had both psychiatric and physical comorbidities, 53.6% at least one psychiatric comorbidity, and 66.5% at least one physical comorbidity. The most prevalent were the essential hypertension (33.5%), various psychoactive substance-related disorders (21.2%), specific personality disorders (14.6%), obesity (14.5%), and disorders of lipoprotein metabolism (14%). The presence of an at least one physical comorbidity, atypical symptoms of BD, and unemployed status were each associated with an at least one psychiatric comorbidity independent of sex, early onset of BD (age of onset <35 years), BD duration and pattern of BD illness progression (*p* < 0.001). The presence of various psychoactive substance-related disorders, BD duration, atypical symptoms of BD, unemployed status, pension, female sex, and not using antipsychotics were each associated with an at least one physical comorbidity independent of the pattern of BD illness progression (*p* < 0.001). In several other multiple regression models, the use of antipsychotics (in particular, olanzapine) was associated with a decreased probability of the essential hypertension and predicted the clinical phenotype of comorbidity-free BD (*p* < 0.05).

**Conclusion:**

This cross-national study has reported novel estimates and clinical correlates related to both the comorbidity-free phenotype and the factors associated with psychiatric and physical comorbidities in adults with BD in Slovakia. The findings provide new insights into understanding of the clinical presentation of BD that can inform clinical practice and further research to continue to investigate potential mechanisms of BD adverse outcomes and disease complications onset.

## 1. Introduction

Bipolar disorder (BD) is a chronic and disabling affective disorder with significant morbidity and mortality. It is characterized by periodic mood dysregulation with persistent mood changes involving elevated mood states (episodes of mania/hypomania or episodes with mixed features) with or without depressed mood states and energy levels (1). According to the most recent Diagnostic and Statistical Manual of Mental Disorders, Fifth Edition, Text Revision (DSM-5-TR) the types of BD include: Bipolar I disorder, Bipolar II disorder, Cyclothymic disorder, Substance/medication induced bipolar disorder, Bipolar disorder due to another medical condition, Other specified bipolar disorder, and Unspecified bipolar disorder (2). Persons with bipolar I disorder have episodes of mania and nearly always experience major depressive episodes. Bipolar II disorder is marked by at least one hypomanic episode, at least one major depressive episode, and the absence of manic episodes.

According to a meta-analysis of 25 population-and community-based epidemiological studies (3), the lifetime prevalence of BD among adults worldwide is approximately 1–2%, with a slight increase in prevalence from the Diagnostic and Statistical Manual of Mental Disorders DSM-III and DSM-IIIR to DSM-IV diagnostic criteria. Bipolar disorder affects males and females at about equivalent rates (4). It is found in all ages, races, ethnic groups and social classes (5). The median age of onset for BD is 25 years, although the illness can start in early childhood or as late as the 40’s and 50’s (5). In recent years, a bimodal (early vs. late) and a trimodal (early vs. mid vs. late) age of onset distribution modalities have been reported for BD, which may help to anticipate disease trajectory and guide appropriate therapeutic decisions (6, 7). Although the peak age of onset is 15–25 years, BD diagnosis and guideline-recommended interventions (e.g., mood stabilizers) are likely to be delayed until age 25–35 (7).

Most persons with BD have at least one comorbid psychiatric or physical illness, and many adults have multiple co-occurring illnesses (8, 9). Research highlights that BD is associated with 2–3 times higher mortality rates; persons affected by BD are expected to live approximately 8.5–9 years less compared to the general population, which is largely due to a high prevalence of physical comorbidity (10). Adults living with BD often experience high levels of weight gain, diabetes, cardiovascular disease and hypertension, which may negatively impact the course of BD (9, 11). Also, it is possible that there is an interaction effect between BD and aging, which may contribute to worse physical health outcomes in older adults with BD (12).

Increasing evidence indicates BD and its shared comorbidity are characterized by similar neuropsychological signatures, implying possibly shared biological pathways. Genome-wide association studies have identified novel shared risk loci with a mixed pattern of effect directions, indicating the presence of the complex genetic overlap between alcohol use behaviors and BD (13). Analyses at not only the genetic level but also the pathway and network level (gene co-expression network analysis of transcriptomic data, contextualization of signals from omics data analyses) help to elucidate the neurobiological underpinnings of BD and its comorbidity. Several dysregulated pathways related to the immune inflammatory system (e.g., TNF, IL-17, and NF-kappa B signaling pathways), nervous system (e.g., dopaminergic and GABAergic synapses) (14), stress response, energy systems, and neuron systems (15) and other risk factors (childhood adversity, diet, physical activity, iatrogenic effects due to medications) might mediate the link between BD and its comorbidity phenotypes. Despite the integrative, data-and knowledge-driven approach to better understand the comorbid associations of BD, the clinical basis and predictors of the BD comorbidity remain only partially explained.

The main objectives of the present nationwide multicenter observational study were to examine, quantify and characterize the cross-sectional associations of psychiatric and physical comorbidities with selected demographic and clinical characteristics in adults with BD. Novel insights may help to clarify the links and the complex interrelationships between the clinical presentation of BD and the associated clinical correlates of BD comorbid conditions. The findings will thus inform clinical practice and further research to continue to investigate potential mechanisms of BD adverse outcomes and disease complications onset.

## 2. Materials and methods

### 2.1. Study design and setting

The study was part of the nationwide project COSMOS (COmorbiditieS in MOst severe neurology & psychiatric disorders in Slovakia) that examined associations of comorbidities of selected neurological and psychiatric disorders (Parkinson’s disease, Alzheimer’s dementia, Depression, Schizophrenia, and Bipolar disorder) with demographic and clinical characteristics.

The present study was a nationwide multicenter cross-sectional observational epidemiological study conducted from October 2015 to March 2017 in Slovakia. The study was approved by the Ethics Committee of the Kosice Self-governing Region (Namestie Maratonu mieru 68/1, 042 66 Kosice, Slovak Republic; study protocol number: 4670/2015/OSVaZ-28576). The research was conducted in accordance with the principles of the Declaration of Helsinki and with the ethical standards of the national committee on human experimentation. All subjects provided written informed consent before participating in the study.

The report of this observational study adheres to the Strengthening the Reporting of Observational Studies in Epidemiology (STROBE) recommendations (16).

### 2.2. Study population

Participants were recruited from 57 outpatient psychiatric facilities across Slovakia. Inclusion criteria were as follows: (i) persons of any sex and gender; (ii) ages 18 years to 80 years; (iii) diagnosis of Bipolar Disorder I or II (International Statistical Classification of Diseases and Related Health Problems 10th Revision ICD-10 Version: 2016 (17)) in any phase of the disorder; (iv) subjects diagnosed with BD at least 2 years prior to study entry; (v) subjects agreeing to participate to study; (vi) provision of subject informed consent (by subjects or by their legal representatives); (vii) subjects who live in Slovakia. Exclusion criteria included subjects not understanding Slovak and subjects with neurological or psychiatric disorders prohibiting their comprehension of the study.

**Figure 1:**
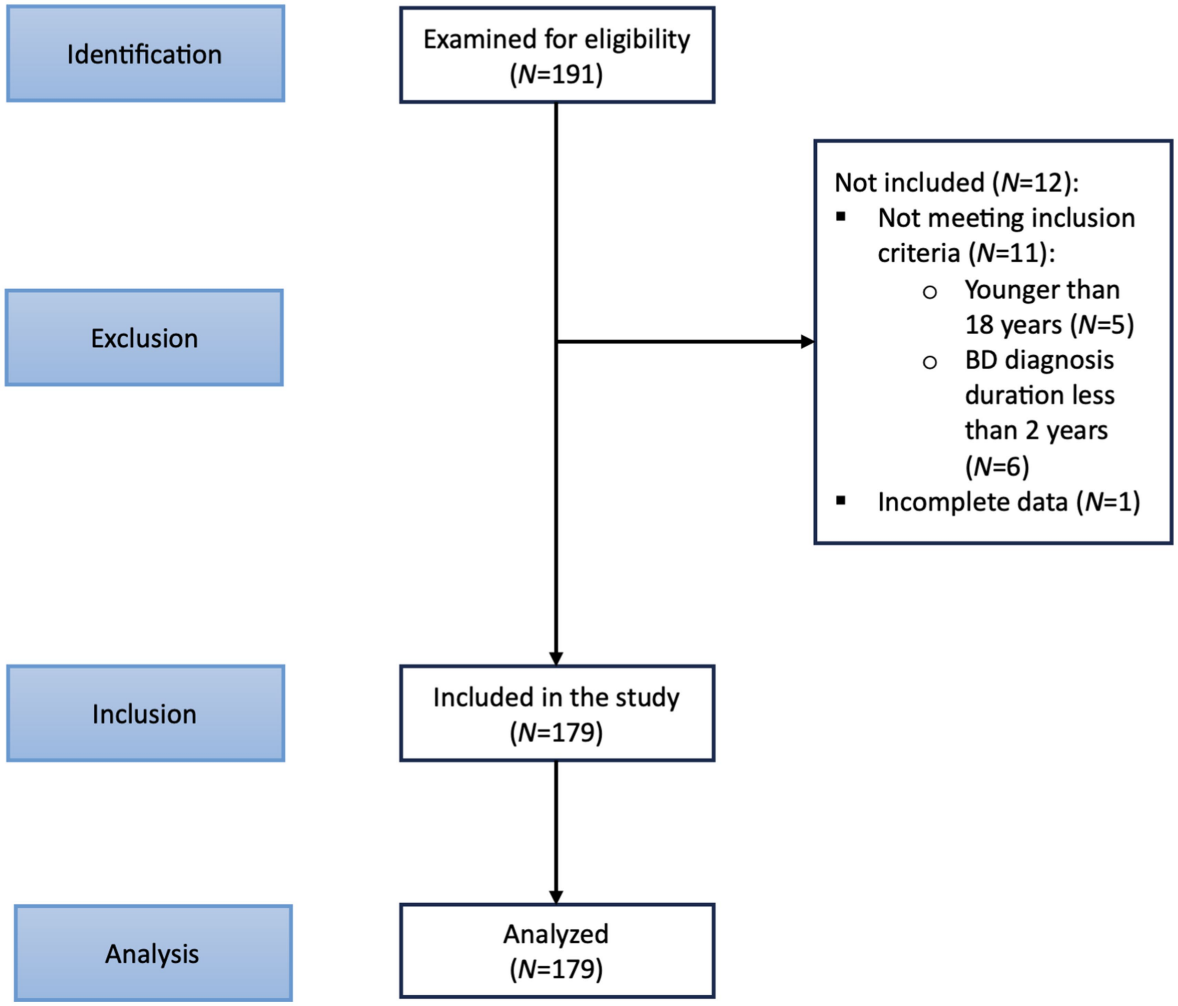
STROBE flow diagram.

Figure 1 depicts the STROBE flow diagram. One hundred ninety-one prospective subjects were initially recruited. During the recruitment visit, study details were discussed with the prospective subjects, and written informed consent was obtained. Recruited subjects were assessed for eligibility through several assessments, including obtaining a medical history. One hundred seventy-nine subjects were identified for inclusion. A specialized platform of the COSMOS study with an online questionnaire was administered to obtain socio-demographic data and clinical information. The presence of comorbid psychiatric and physical conditions was determined utilizing information from medical records.

### 2.3. Outcome measures

The primary outcome measures included the prevalence and factors associated with psychiatric and physical comorbidities in BD. The secondary outcome measures were as follows: selected demographic variables, BD historical illness variables - BD diagnosis duration, current stage of BD, clinical evolution/illness progression of BD, number of previous crises/bipolar mood episodes (defined as an episode of bipolar depression or mania/hypomania or mixed) in the past 2 years prior to study entry, types of current psychopharmacological treatment provided (monotherapy vs. combination treatment), estimation of bipolar medication adherence in the past 2 years prior to study entry, efficacy of psychopharmacological treatment, interval of psychiatric follow-up appointments in the past 2 years prior to study entry, functional impairment/disability, employment status, and clinical phenotype of comorbidity-free BD.

### 2.4. Statistical analyses

The Shapiro–Wilk test was applied to test for a normal distribution. Categorical data are presented as absolute and relative counts. Interval data are presented as medians and interquartile ranges (IQR). A comparison of interval variables between two groups has been performed by the parametric Student t-test and by the non-parametric Wilcoxon rank-sum test, where applicable. The chi-squared test was used to examine patterns between categorical variables. Subgroup analyses by sex and age of onset (specified *a priori*) were performed to assess the subgroup-specific potential differences in BD characteristics and factors associated with comorbid conditions. Our sample’s cutoff age of onset for BD was 35 years (6, 7).

Both correlation and simple linear regression were used to examine the presence of a linear relationship between two variables. Among continuous variables, only age was normally distributed. Therefore, Spearman’s rank correlation coefficient (rho) was utilized to examine the relationship between two independent variables in a sample. The relationship between dependent/outcome variables and their predictors was explored by linear regression for interval dependent variables and logistic regression for categorical dependent variables. The postestimation marginal means, predictive margins, and marginal effects were computed. The prediction graphs were created to reflect the adjusted predicted probabilities of the dependent variables of interest along with their 95% confidence intervals (CI) for the significant continuous outcomes and for each group of factor variables.

Besides the stratification, multiple regression analyses were performed to determine whether unadjusted associations persisted after controlling for potential confounders. When building multiple regression prediction models, we had considered: (i) importance of the variable, (ii) variables based on causal understanding (using the disjunctive cause criterion (18)), and (iii) interaction terms of variables with large main effects. For each of the independent variables/covariates included in the regression models, we checked for the presence of multicollinearity. Variance inflation factors for the independent variables specified in a linear regression model were calculated. If the reciprocal of the uncentered variance inflation factor was smaller than the predetermined tolerance, the associated predictor variable was removed from the regression model.

Findings were considered to be statistically significant at the 5% level. Statistical analyses were performed using Stata Special Edition statistical software Version 13.1 (StataCorp LP, College Station, TX).

## 3. Results

### 3.1. Sample characteristics and univariate analyses

**Figure 2:**
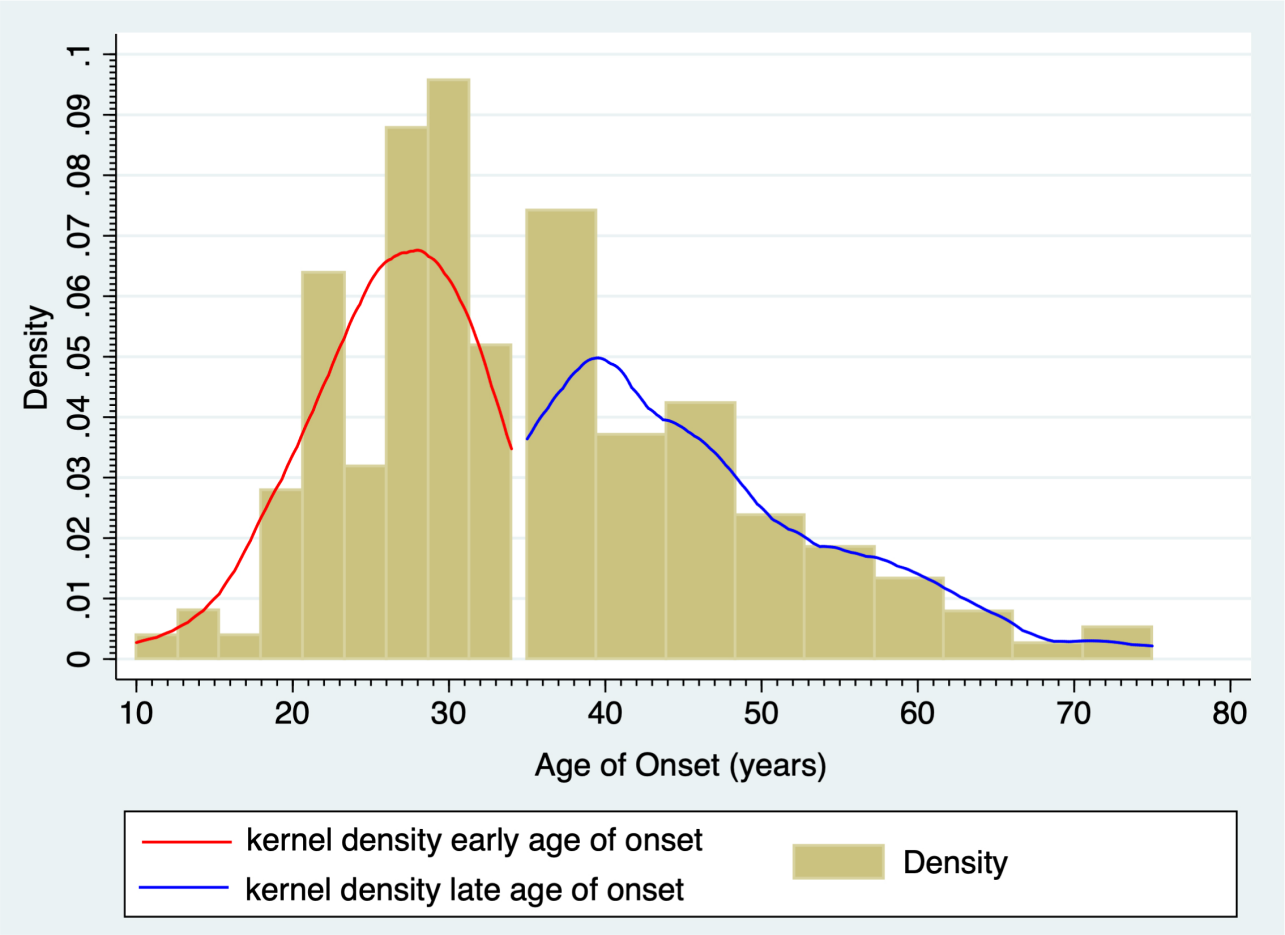
The bimodal age of onset distribution modality for bipolar disorder.

The study sample comprised 179 enrolled participants who met inclusion criteria, of which 103 (57.5%) were females. The early-onset subgroup included 94 (52.5%) individuals with BD age of onset <35 years. The late-onset subgroup comprised 85 (47.5%) subjects with BD age of onset ≥35 years. Figure 2 shows the bimodal age of onset distribution modality for BD. Table 1 shows demographics and BD illness characteristics of the study participants and subgroups by sex and age of onset of BD. The median (IQR) age of onset was 27 (19–26) years in the early-onset subgroup and 44 (27–41) years in the late-onset subgroup (*p* < 0.001). Subjects with the early onset of BD were younger compared to those with the late onset of BD (*p* < 0.001).

**Table 1:** Demographics and bipolar disorder illness characteristics of study participants and subgroups by sex and age of onset of bipolar disorder.

Variable	All (*N* = 179)	Females (*N* = 103)	Males (*N* = 76)	*p*-value	Early-onset^#^ (*N* = 94)	Late-onset^#^ (*N* = 85)	*p*-value
Age, years	49 (38–58)	50 (38–60)	47 (38–55)	0.309	39 (33–49)	55 (49–64)	<0.001
Age of onset, years	34 (27–43)	32 (26–45)	34 (27.5–41.5)	0.514	27 (23–30)	44 (37–51)	**<0.001**
BD diagnosis duration, years	10 (6–19)	10 (5–19)	11.5 (6–17.5)	0.549	12 (7–22)	10 (5–15)	**<0.001**
*Rapid cycling*							
Yes	22 (12.3)	15 (8.4)	7 (3.9)	0.281	9 (5)	13 (7.3)	0.244
No	157 (87.7)	88 (49.2)	69 (38.6)		85 (47.5)	72 (40.2)	
*Seasonal pattern*							
Yes	74 (41.3)	43 (24)	31 (17.3)	0.898	29 (16.2)	45 (25.1)	**0.003**
No	105 (58.7)	60 (33.5)	45 (25.1)		65 (36.3)	40 (22.4)	
*Atypical symptoms*							
Yes	42 (23.5)	26 (14.5)	16 (8.9)	0.513	20 (11.2)	22 (12.3)	0.468
No	137 (76.5)	77 (43)	60 (33.5)		74 (41.3)	63 (35.2)	
*Number of mood episodes* *							
Bipolar depression	2 (1–2)	2 (1–3)	1 (1–2)	*0.053*	2 (1–2)	2 (1–3)	0.211
Mania/hypomania	1 (1–2)	1 (1–2)	1 (1–1)	0.163	1 (1–1)	1 (1–2)	**0.022**
Mixed	1 (1–2)	1 (1–2)	1 (1–2)	0.787	1 (1–2)	1 (1–2)	0.249
*Current psychopharmacotherapy*							
Monotherapy	35 (19.6)	21 (11.7)	14 (7.8)	0.743	16 (8.9)	19 (10.6)	0.369
Combination treatment	144 (80.4)	82 (45.8)	62 (34.6)		78 (43.6)	66 (36.9)	
Antipsychotics	86 (48)	49 (27.3)	37 (20.7)	0.883	45 (25.1)	41 (22.9)	0.961
*The most commonly used mood stabilizers and antipsychotics*							
Valproate	78 (43.6)	43 (24)	35 (19.6)	0.566	45 (25.2)	33 (18.4)	0.223
Lithium	33 (18.4)	14 (7.8)	19 (10.6)	*0.052*	19 (10.6)	14 (7.8)	0.519
Quetiapine	82 (45.8)	47 (26.3)	35 (19.6)	0.955	49 (27.4)	33 (18.4)	0.074
Olanzapine	34 (19)	19 (10.6)	15 (8.4)	0.828	15 (8.4)	19 (10.6)	0.276
Aripiprazole	14 (7.8)	5 (2.8)	9 (5)	*0.085*	11 (6.1)	3 (1.7)	**0.042**
Haloperidol	10 (5.6)	6 (3.4)	4 (2.2)	0.871	5 (2.8)	5 (2.8)	0.870
Chlorprothixene	10 (5.6)	7 (3.9)	3 (1.7)	0.412	6 (3.4)	4 (2.2)	0.626
Flupentixol	7 (3.9)	3 (1.7)	4 (2.2)	0.423	4 (2.2)	3 (1.7)	0.802
Zuclopenthixol	6 (3.4)	3 (1.7)	3 (1.7)	0.704	4 (2.2)	2 (1.12)	0.480
Asenapine	6 (3.4)	2 (1.12)	4 (2.2)	0.222	4 (2.2)	2 (1.12)	0.480
Tiapride	6 (3.4)	4 (2.2)	2 (1.12)	0.646	0 (0)	6 (3.4)	**0.009**
*BD medication adherence*							
Low	2 (1.12)	1 (0.56)	1 (0.56)	0.989	0 (0)	2 (1.12)	**0.022**
Low-moderate	12 (6.7)	7 (3.9)	5 (2.8)		9 (5)	3 (1.7)	
Moderate	97 (54.2)	55 (30.7)	42 (23.5)		43 (24)	54 (30.2)	
High	68 (38)	40 (22.3)	28 (15.6)		42 (23.5)	26 (14.5)	
*Efficacy of psychopharmacotherapy*							
High	146 (81.6)	82 (45.8)	64 (35.8)	0.433	77 (43)	69 (38.6)	0.899
Low	33 (18.4)	21 (11.7)	12 (6.7)		17 (9.5)	16 (8.9)	
*Interval of psychiatric follow-up appointments*							
Weekly	20 (11.2)	10 (5.6)	10 (5.6)	0.298	12 (6.7)	8 (4.5)	0.326
Monthly	119 (66.5)	72 (40.2)	47 (26.3)		66 (36.9)	53 (29.6)	
Quarterly	38 (21.2)	21 (11.7)	17 (9.5)		15 (8.4)	23 (12.9)	
Yearly	2 (1.12)	0 (0)	2 (1.12)		1 (0.56)	1 (0.56)	

Data expressed as *N* (%) or median (interquartile range). *N*, number; BD, bipolar disorder. The late-onset subgroup comprised subjects with age of onset ≥ 35 years.

* In the past 2 years prior to study entry.

# The early-onset subgroup included individuals with age of onset < 35 years.The italic values indicate the presence of the trend (*p*<0.1).The bold values reflect the presence of statistical significance (*p*<0.05).

The median (IQR) time since BD diagnosis was 10 (6–18, 42) years in the entire sample. Seasonal pattern of BD was more common in the late-onset compared to the early-onset subgroup (25.1% vs. 16.2%, respectively; *p* = 0.003). As for the number of crises/bipolar mood episodes in the past 2 years prior to study entry, significantly more manic/hypomanic episodes were reported for the late-onset subgroup (*p* = 0.022). The estimated probability of this outcome was 0.606. In addition, there was a trend towards more frequent bipolar depressive episodes in females compared to males (*p* = 0.053).

A majority (80.4%) of subjects were on a combination treatment of BD. In our study, the most commonly used mood stabilizers were valproate (43.6%) and lithium (18.4%). Antipsychotics were used in 48% of the study participants (27.3% females, 20.7% males). The most commonly used antipsychotics were quetiapine (45.8%), olanzapine (19%), and aripiprazole (7.8%), followed by haloperidol (5.6%), chlorprothixene (5.6%), flupentixol (3.9%), zuclopenthixol (3.4%), asenapine (3.4%), tiapride (3.4%), and others. The most commonly used antidepressants were selective serotonin reuptake inhibitors (SSRIs; 27.4%) and selective serotonin and norepinephrine reuptake inhibitors (SNRIs; 20.1%), followed by atypical antidepressants (11.2%), serotonin receptor antagonists and reuptake inhibitors (SARIs; 3.4%), and tricyclic antidepressants (TCAs; 2.2%). Significantly more study participants with the early onset of BD were treated with aripiprazole compared to the late-onset subgroup (*p* = 0.042). Also, there was a trend towards a more frequent use of quetiapine in the early-onset subgroup (*p* = 0.074). Only subjects with the late onset of BD were treated with tiapride (*p* = 0.009). With respect to sex differences in psychopharmacotherapy, there were trends towards a more frequent use of lithium (*p* = 0.052) and aripiprazole (*p* = 0.085) in males compared to females with BD. Significantly higher bipolar medication adherence was apparent in the early-onset subgroup compared with the late-onset subgroup (“high” adherence: 25.2% vs. 18.4%, respectively; *p* = 0.022).

As regards the employment status, 38.6% of the study participants were not employed, 24.6% had full-time employment, 17.9% were pensioners, 13.4% had part-time employment, and 5.6% casual employment. Functional impairment/disability was reported in 51.4% of the subjects. The majority (48%) were disabled mainly due to a psychiatric condition. Only 3.4% of the sample were disabled mainly due to a non-psychiatric condition. While there were no sex differences in the employment status, there were more unemployed individuals in the early-onset compared with the late-onset subgroup (25.1% vs. 13.4%, respectively). The late-onset subgroup had more pensioners compared to the early-onset subgroup (14% vs. 3.9%, respectively; *p* = 0.001).

**Figure 3:**
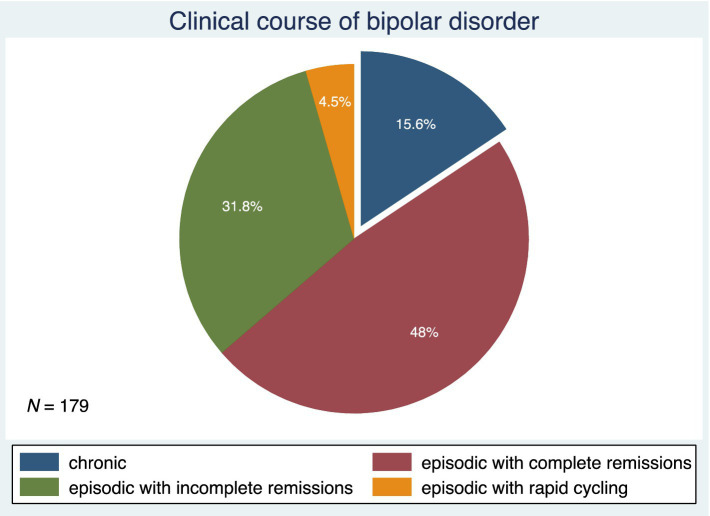
The patterns of bipolar disorder illness progression, *N*, number.

**Figure 4:**
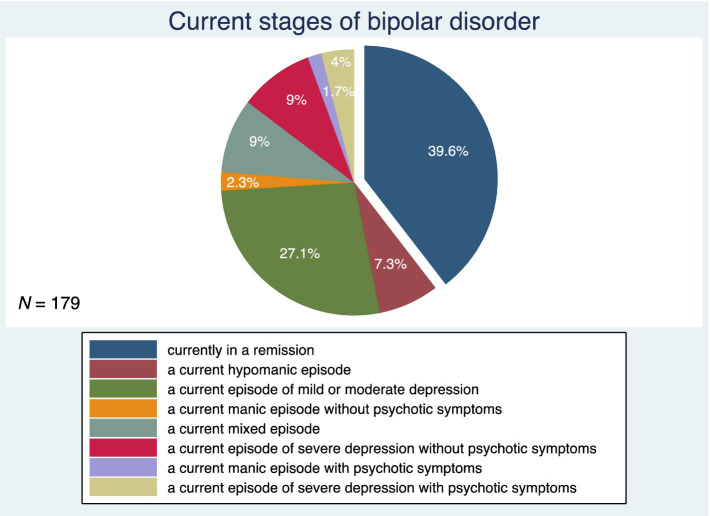
Current stages of bipolar disorder, *N*, number.

Figure 3 shows the patterns of BD illness progression. Forty-eight percent of subjects had an episodic course of BD with complete remissions. This was followed by an episodic course of BD with incomplete remissions in 31.8%, a chronic course of BD in 15.6%, and an episodic course of BD with rapid cycling in 4.5% of the study participants. Figure 4 depicts the current stages of BD in the sample. Most of the subjects (39.6%) were currently in remission. A current episode of mild or moderate depression was present in 27.1% of the study participants, and a current episode of severe depression without psychotic symptoms was present in 9%. Similarly, 9% of individuals were in a current mixed episode.

The comorbidity-free sample of study participants with BD comprised 19 females (10.6%) and 21 males (11.8%); *p* = 0.145. The number of individuals without comorbidities was equally distributed between the early-onset and late-onset subgroups [20 (11.2%) vs. 20 (11.2%), respectively; *p* = 0.718]. Seventy-six (42.5%) subjects [49 females (27.4%) and 27 males (15.1%); *p* = 0.107] had both psychiatric and physical comorbidities. The number of individuals with both psychiatric and physical comorbidities was similar in the early-onset compared to the late-onset subgroup [36 (20.1%) vs. 40 (22.4%), respectively; *p* = 0.236].

Table 2 shows the distribution of psychiatric comorbidities of BD in all study participants and in subgroups by sex and age of onset of BD. The psychiatric conditions in the table are listed by groups based on 2023 ICD-10-CM Codes (42). Nearly 54% of all study participants had at least one psychiatric comorbidity. The most prevalent were the various psychoactive substance-related disorders (21.2%), followed by specific personality disorders (14.6%), in particular, the borderline personality disorder (7.3%), the adjustment disorder with anxiety (6.7%), sleep disorders (6.2%), organic non-psychotic disorders (5.6%), eating disorders (3.4%), and other psychiatric disorders (3.4%). Two psychiatric comorbidities were present in 11.2%, three psychiatric comorbidities in 2.2%, and four psychiatric comorbid conditions in 1.7% of the study participants. Three or more psychiatric comorbidities were more likely to occur in persons with the early onset of BD (*p* = 0.073). Compared to males, females had significantly higher prevalences of sleep disorders (*p* = 0.021) and eating disorders (*p* = 0.032). There was a trend towards a more frequent occurrence of the adjustment disorder with anxiety in females than in males with BD (*p* = 0.061). The late-onset subgroup had a higher prevalence of the adjustment disorder with anxiety compared with the early-onset subgroup (*p* = 0.048). Finally, there was a trend towards a more prevalent sexual dysfunction in the early-onset subgroup (*p* = 0.054).

**Table 2:** Psychiatric comorbidities of bipolar disorder in all study participants and in subgroups by sex and age of onset.

Variable	All (*N* = 179)	Females (*N* = 103)	Males (*N* = 76)	*p*-value	Early-onset^#^ (*N* = 94)	Late-onset^#^ (*N* = 85)	*p*-value
At least one psychiatric comorbidity	96 (53.6)	56 (31.3)	40 (22.3)	0.818	50 (27.9)	46 (25.7)	0.901
One psychiatric comorbidity	69 (38.6)	38 (21.2)	31 (17.3)	0.597	33 (18.4)	36 (20.1)	0.320
At least two psychiatric comorbidities	27 (15.1)	18 (10.1)	9 (5)	0.298	17 (9.5)	10 (5.6)	0.238
Two psychiatric comorbidities	20 (11.2)	13 (7.3)	7 (3.9)	0.474	11 (6.2)	9 (5)	0.813
At least three psychiatric comorbidities	7 (3.9)	5 (2.8)	2 (1.12)	0.448	6 (3.4)	1 (0.56)	*0.073*
Three psychiatric comorbidities	4 (2.2)	2 (1.12)	2 (1.12)	0.758	3 (1.7)	1 (0.56)	0.362
Four psychiatric comorbidities	3 (1.7)	3 (1.7)	0 (0)	0.134	3 (1.7)	0 (0)	*0.097*
Mental disorders due to known physiological conditions (F01–F09)*							
Dementia (F01, F02, F03)	0 (0)	0 (0)	0 (0)	-	0 (0)	0 (0)	-
Organic psychotic disorders	0 (0)	0 (0)	0 (0)	-	0 (0)	0 (0)	-
Organic non-psychotic disorders	10 (5.6)	6 (3.4)	4 (2.2)	0.871	3 (1.7)	7 (3.9)	0.142
Mental and behavioral disorders due to psychoactive substance use (F10–F19)*							
Various psychoactive substance-related disorders	38 (21.2)	18 (10.1)	20 (11.2)	0.153	22 (12.3)	**16 (8.9)**	0.454
Schizophrenia, schizotypal, delusional, and other non-mood psychotic disorders (F20–F29)*							
Psychotic disorders from the schizophrenia spectrum	4 (2.2)	2 (1.12)	2 (1.12)	0.758	3 (1.7)	1 (0.56)	0.362
Anxiety, dissociative, stress-related, somatoform and other nonpsychotic mental disorders (F40–F48)*							
Panic disorder	3 (1.7)	2 (1.12)	1 (0.56)	0.747	2 (1.12)	1 (0.56)	0.621
Generalized anxiety disorder	4 (2.2)	3 (1.7)	1 (0.56)	0.475	2 (1.12)	2 (1.12)	0.919
Adjustment disorder with anxiety	12 (6.7)	10 (5.6)	2 (1.12)	*0.061*	3 (1.7)	9 (5)	**0.048**
Post-traumatic stress disorder	5 (2.8)	3 (1.7)	2 (1.12)	0.910	3 (1.7)	2 (1.12)	0.734
Behavioral syndromes associated with physiological disturbances and physical factors (F50–F59)*							
Eating disorders	6 (3.4)	6 (3.4)	0 (0)	**0.032**	5 (2.8)	1 (0.56)	0.124
Sleep disorders	11 (6.2)	10 (5.6)	1 (0.56)	**0.021**	7 (3.9)	4 (2.2)	0.446
Sexual dysfunction	4 (2.2)	2 (1.12)	2 (1.12)	0.758	4 (2.2)	0 (0)	*0.054*
Disorders of adult personality and behavior (F60–F69)*							
Borderline personality disorder (F60.3)	13 (7.3)	8 (4.5)	5 (2.8)	0.762	7 (3.9)	6 (3.4)	0.920
Specific personality disorders other than F60.3	13 (7.3)	7 (3.9)	6 (3.4)	0.780	9 (5)	4 (2.2)	0.210
Behavioral and emotional disorders with onset usually occurring in childhood and adolescence (F90–F98)*							
Attention-deficit hyperactivity disorders	4 (2.2)	2 (1.12)	2 (1.12)	0.758	3 (1.7)	1 (0.56)	0.362
*Other*							
Other psychiatric disorders	6 (3.4)	3 (1.7)	3 (1.7)	0.704	3 (1.7)	3 (1.7)	0.900

Data expressed as N (%). *N*, number. The late-onset subgroup comprised subjects with age of onset ≥ 35 years.

* 2023 ICD-10-CM Codes (42).

# The early-onset subgroup included individuals with age of onset < 35 years.The italic values indicate the presence of the trend (*p*<0.1). The bold values reflect the presence of statistical significance (*p*<0.05).

Table 3 shows the comorbid physical conditions in all study participants and in subgroups by sex and age of onset of BD. The conditions are listed by groups based on the 2023 ICD-10-CM Codes (42). More than 66% of all study participants had at least one physical comorbidity, and females were more affected than males (*p* = 0.006). Compared to males, females with BD had higher prevalences of thyroid gland disorders (*p* = 0.005) and various diseases of the musculoskeletal system and connective tissue (*p* = 0.020). In addition, there were trends towards higher prevalences of malignant neoplasms (*p* = 0.051), various diseases of blood and blood-forming organs (*p* = 0.051), osteoporosis (*p* = 0.093), headache (*p* = 0.066), and other physical comorbidities (*p* = 0.051) in females compared to males. The seven most prevalent physical comorbidities of BD in our sample comprised the essential (primary) hypertension (33.5%), obesity (14.5%), disorders of lipoprotein metabolism and other lipidemias (14%), chronic ischemic heart disease (11.2%), various other diseases of the musculoskeletal system and connective tissue (10.1%), headache (8.4%), and disorders of thyroid gland (7.8%). At least one cardiovascular disease occurred in 69 (38.6%) of the study participants. At least 1 endocrine/nutritional/metabolic disease was present in 56 (31.3%) of subjects. Interestingly, a significantly higher prevalence of the cardiovascular but not metabolic comorbidities was observed in subjects with the late onset of BD. Specifically, significantly higher prevalences of the essential (primary) hypertension (*p* = 0.001), chronic ischemic heart disease (*p* = 0.002), and peripheral vascular disease (*p* = 0.006) were apparent in the late-onset subgroup compared to the early-onset subgroup. In addition, the late-onset subgroup had higher prevalences of osteoporosis (*p* < 0.001), headache (*p* = 0.036), urinary incontinence (*p* = 0.033), various other diseases of the genitourinary system (*p* = 0.017), along with a trend towards more prevalent various other chronic respiratory diseases (*p* = 0.066).

**Table 3:** Comorbid physical conditions in all study participants and in subgroups by sex and age of onset of bipolar disorder.

Variable	All (*N* = 179)	Females (*N* = 103)	Males (*N* = 76)	*p*-value	Early-onset^#^ (*N* = 94)	Late-onset^#^ (*N* = 85)	*p*-value
At least one physical comorbidity	119 (66.5)	77 (43)	42 (23.5)	**0.006**	60 (33.5)	59 (33)	0.430
Certain infectious and parasitic diseases (A00–B99)*							
Chronic infectious diseases	2 (1.12)	2 (1.12)	0 (0)	0.222	2 (1.12)	0 (0)	0.176
Neoplasms (C00-D49)*							
Malignant neoplasms	5 (2.8)	5 (2.8)	0 (0)	*0.051*	1 (0.56)	4 (2.2)	0.140
Diseases of the blood and blood-forming organs (D50–D89)*							
Various diseases of blood and blood-forming organs	5 (2.8)	5 (2.8)	0 (0)	*0.051*	3 (1.7)	2 (1.12)	0.734
Endocrine, nutritional and metabolic diseases (E00–E89)*							
At least 1 endocrine, nutritional or metabolic disease	56 (31.3)	37 (20.7)	19 (10.6)	0.119	30 (16.8)	26 (14.5)	0.848
Obesity, unspecified	26 (14.5)	18 (10)	8 (4.5)	0.192	14 (7.8)	12 (6.7)	0.883
Disorders of lipoprotein metabolism and other lipidemias	25 (14)	16 (9)	9 (5)	0.481	10 (5.6)	15 (8.4)	0.177
Disorders of thyroid gland	14 (7.8)	13 (7.3)	1 (0.56)	**0.005**	7 (3.9)	7 (3.9)	0.844
Type 1 diabetes mellitus	1 (0.56)	1 (0.56)	0 (0)	0.389	0 (0)	1 (0.56)	0.292
Type 2 diabetes mellitus	10 (5.6)	6 (3.4)	4 (2.2)	0.871	5 (2.8)	5 (2.8)	0.870
Hyperprolactinemia	1 (0.56)	1 (0.56)	0 (0)	0.389	1 (0.56)	0 (0)	0.340
Diseases of the nervous system (G00–G99)*							
Epilepsy and recurrent seizures	6 (3.4)	4 (2.2)	2 (1.12)	0.646	2 (1.12)	4 (2.2)	0.339
Cerebral infarction, unspecified	4 (2.2)	2 (1.12)	2 (1.12)	0.758	2 (1.12)	2 (1.12)	0.919
Various other neurological diseases	11 (6.2)	6 (3.4)	5 (2.8)	0.836	5 (2.8)	6 (3.4)	0.628
Diseases of the circulatory system (I00–I99)*							
At least one cardiovascular disease	69 (38.6)	41 (22.9)	28 (15.7)	0.687	26 (14.6)	43 (24)	**0.002**
Essential (primary) hypertension	60 (33.5)	39 (21.8)	21 (11.7)	0.152	21 (11.7)	39 (21.8)	**0.001**
Chronic ischemic heart disease	20 (11.2)	13 (7.3)	7 (3.9)	0.474	4 (2.2)	16 (9)	**0.002**
Cardiac arrhythmias	6 (3.4)	4 (2.2)	2 (1.12)	0.646	2 (1.12)	4 (2.2)	0.339
Peripheral vascular disease	10 (5.6)	8 (4.5)	2 (1.12)	0.139	1 (0.56)	9 (5)	**0.006**
Various other cardiovascular diseases	3 (1.7)	1 (0.56)	2 (1.12)	0.392	1 (0.56)	2 (1.12)	0.502
Diseases of the respiratory system (J00–J99)*							
Asthma	3 (1.7)	2 (1.12)	1 (0.56)	0.747	1 (0.56)	2 (1.12)	0.502
Various other chronic respiratory diseases	3 (1.7)	2 (1.12)	1 (0.56)	0.747	0 (0)	3 (1.7)	*0.066*
Diseases of the digestive system (K00–K95)*							
Hepatic failure	1 (0.56)	1 (0.56)	0 (0)	0.389	1 (0.56)	0 (0)	0.340
Other specified diseases of the digestive system	11 (6.2)	7 (3.9)	4 (2.2)	0.673	5 (2.8)	6 (3.4)	0.628
Diseases of the musculoskeletal system and connective tissue (M00–M99)*							
Osteoporosis, unspecified	11 (6.1)	9 (5)	2 (1.12)	*0.093*	0 (0)	11 (6.1)	**<0.001**
Various other diseases of the musculoskeletal system and connective tissue	18 (10.1)	15 (8.4)	3 (1.7)	**0.020**	7 (3.9)	11 (6.1)	0.222
Diseases of the genitourinary system (N00–N99)*							
Unspecified urinary incontinence	4 (2.2)	3 (1.7)	1 (0.56)	0.475	0 (0)	4 (2.2)	**0.033**
Various renal diseases	3 (1.7)	2 (1.12)	1 (0.56)	0.747	1 (0.56)	2 (1.12)	0.502
Various other diseases of the genitourinary system	5 (2.8)	2 (1.12)	3 (1.7)	0.421	0 (0)	5 (2.8)	**0.017**
*Other*							
Headache	15 (8.4)	12 (6.7)	3 (1.7)	*0.066*	4 (2.2)	11 (6.2)	**0.036**
Other physical comorbidities	5 (2.8)	5 (2.8)	0 (0)	*0.051*	3 (1.7)	2 (1.12)	0.734

Data expressed as N (%). *N*, number. The late-onset subgroup comprised subjects with age of onset ≥ 35 years.

* 2023 ICD-10-CM Codes (42).

# The early-onset subgroup included individuals with age of onset < 35 years.The italic values indicate the presence of the trend (*p*<0.1).The bold values reflect the presence of statistical significance (*p*<0.05).

### 3.2. Bivariate analyses

**Figure 5:**
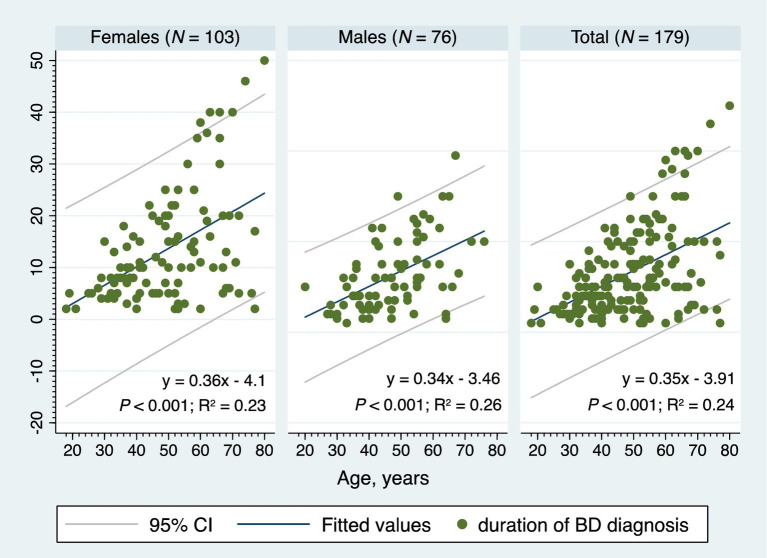
The two-way linear prediction plot for the bipolar disorder diagnosis duration, BD, bipolar disorder, 95% CI, 95% confidence interval, *N*, number.

Significant positive correlations were found between the age and the age of onset (rho = 0.70; *p* < 0.001) and between the age and the duration of BD diagnosis (rho = 0.46; *p* < 0.001). The age of onset and the duration of BD diagnosis were negatively correlated (rho = −0.20; *p* = 0.006). The number of manic/hypomanic episodes in the past 2 years prior to study entry increased proportionally with the increasing age of onset (rho = 0.25; *p* = 0.013). Positive associations were observed among different types of bipolar mood episodes in the past 2 years prior to study entry; the more previous depressive episodes, the more previous mixed episodes (rho = 0.45; *p* < 0.001), and the more previous depressive episodes, the more previous manic/hypomanic crises (rho = 0.32; *p* = 0.002). Figure 5 shows the two-way linear prediction plot (for females, males, total) for the duration of BD diagnosis calculated from a linear regression of the duration of BD diagnosis on age. The resulting line is plotted along with a 95% confidence interval based on the standard error of the forecast.

**Figure 6:**
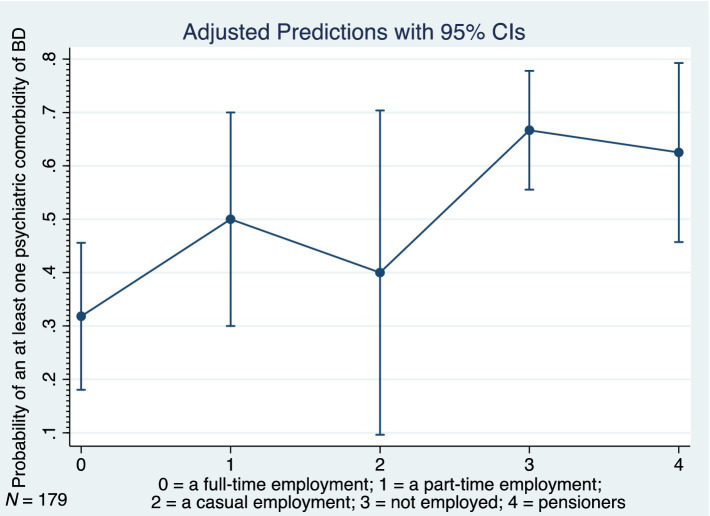
Adjusted predictions of employment status for probability of at least one psychiatric comorbidity in bipolar disorder, BD, bipolar disorder, 95% CI, 95% confidence interval, *N*, number.

Table 4 shows the relations between at least one psychiatric comorbidity of BD and its associated factors (only the significant results and trends are listed). The logistic regression analyses were performed on the whole cohort and on subgroups based on a bimodal age of onset distribution modality (early-onset vs. late-onset BD). Similarly, the comparisons were performed between males and females. In our study, the strongest predictor of an at least one psychiatric comorbidity of BD was the presence of an at least one physical comorbidity in all (*p* < 0.001), in females (*p* = 0.002), in males (*p* = 0.025), and particularly in those with the late-onset BD (*p* < 0.001). The findings suggest that the odds of an at least one psychiatric comorbidity of BD (in all) is 3.54 times higher given at least one physical comorbidity diagnosis compared to no physical comorbidity. The odds ratios (OR), 95% confidence intervals (95% CI) and *p*-values of the significant physical comorbidities are listed in Table 4. As for the clinical phenotype of BD, the atypical symptoms of BD (particularly in females), rapid cycling in females, current hypomanic episode in all, current episode of mild or moderate depression in all, and the longer duration of late-onset BD each significantly increased occurrence of an at least one psychiatric comorbidity of BD (Table 4). The seasonal pattern of BD in late-onset BD decreased probability of the psychiatric comorbidity (OR 0.29; 95% CI: 0.116–0.704; *p* = 0.006). The higher odds of an at least one psychiatric comorbidity of BD had those who were unemployed (OR 4.29; 95% CI: 1.91–9.615; *p* < 0.001), pensioners (OR 3.57; 95% CI: 1.373–9.294; *p* = 0.009) or were disabled mainly due to a psychiatric condition (OR 2.07; 95% CI: 1.129–3.8; *p* = 0.019). The subgroup analyses confirmed the statistical significance of the unemployed status to predict at least one psychiatric comorbidity in females, males, early-onset, and late-onset BD. Figure 6 depicts the adjusted predictions of employment status for the probability of an at least one psychiatric comorbidity in BD. The graph shows the computed predicted probability of an at least one psychiatric comorbidity of BD in each of the employment status groups along with their 95% CIs.

**Table 4:** Factors associated with an at least one psychiatric comorbidity of bipolar disorder (only the significant results and trends are listed).

	*N*	Odds ratio	Standard error	95% confidence interval	*p*-value
*Binary predictors*					
Combination treatment in males	76	3.46	2.233	0.978–12.259	*0.054*
Combination treatment in early-onset BD	94	3	1.758	0.951–9.461	*0.061*
Atypical symptoms in all	179	3.65	1.463	1.664–8.005	**0.001**
Atypical symptoms in females	103	3.80	1.968	1.374–10.488	**0.010**
Atypical symptoms in males	76	3.43	2.169	0.992–11.848	*0.051*
Seasonal pattern in late-onset BD	85	0.29	0.131	0.116–0.704	**0.006**
Rapid cycling in all	179	2.57	1.295	0.955–6.901	*0.062*
Rapid cycling in females	103	4	2.719	1.055–15.16	**0.041**
At least one physical comorbidity in all	179	3.54	1.18	1.838–6.8	**< 0.001**
At least one physical comorbidity in females	103	4.75	2.383	1.777–12.696	**0.002**
At least one physical comorbidity in males	76	2.91	1.389	1.14–7.417	**0.025**
At least one physical comorbidity in early-onset BD	94	2.14	0.936	0.91–5.045	*0.081*
At least one physical comorbidity in late-onset BD	85	7.02	3.807	2.423–20.322	**< 0.001**
Essential (primary) hypertension in all	179	2.81	0.945	1.452–5.432	**0.002**
Essential (primary) hypertension in females	103	3.97	1.771	1.659–9.517	**0.002**
Essential (primary) hypertension in late-onset BD	85	3.96	1.848	1.586–9.885	**0.003**
Obesity in late-onset BD	85	5.14	4.158	1.052–25.099	**0.043**
Disorders of lipoprotein metabolism and other lipidemias in all	179	3.17	1.568	1.2–8.359	**0.020**
Disorders of lipoprotein metabolism and other lipidemias in females	103	4.43	2.995	1.18–16.663	**0.027**
Disorders of lipoprotein metabolism and other lipidemias in late-onset BD	85	7.29	5.804	1.53–34.714	**0.013**
Various other diseases of the musculoskeletal system and connective tissue in all	179	3.37	1.984	1.064–10.685	**0.039**
Headache in all	179	6.34	4.919	1.388–29	**0.017**
Headache in females	103	4.89	3.925	1.015–23.577	**0.048**
Headache in late-onset BD	85	4.5	3.67	0.91–22.255	*0.065*
Other specified diseases of the digestive system in all	179	9.54	10.108	1.194–76.151	**0.033**
Peripheral vascular disease in late-onset BD	85	8	8.682	0.954–67.113	*0.055*
*Continuous predictors*					
Age in late-onset BD	85	1.04	0.024	0.998–1.093	*0.064*
BD diagnosis duration in late-onset BD	85	1.09	0.042	1.013–1.177	**0.022**
Number of mixed episodes in all*	65	2.78	1.652	0.864–8.911	*0.086*
Number of mixed episodes in males	23	8	9.502	0.78–82.053	*0.080*
Number of mixed episodes in late-onset BD	38	6.9	7.94	0.724–65.799	*0.093*
*Factor variables*					
Current stage of BD in all	177				
Currently in a remission		A reference category			
A current hypomanic episode		4.19	2.939	1.062–16.564	**0.041**
A current episode of mild or moderate depression		2.29	0.886	1.077–4.888	**0.031**
A current manic episode without psychotic symptoms		1.26	1.294	0.168–9.445	0.823
A current mixed episode		0.98	0.546	0.328–2.924	0.969
A current episode of severe depression without psychotic symptoms		2.78	1.635	0.87–8.807	*0.085*
A current manic episode with psychotic symptoms		0.63	0.785	0.055–7.263	0.710
A current episode of severe depression with psychotic symptoms		0.50	0.438	0.091–2.772	0.430
*Employment status*	179				
A full-time employment		A reference category			
A part-time employment		2.14	1.116	0.772–5.949	0.144
A casual employment		1.43	1.032	0.347–5.882	0.621
Not employed		4.29	1.767	1.91–9.615	**< 0.001**
Pensioners		3.57	1.743	1.373–9.294	**0.009**
*Permanent disability*	179				
No disability		A reference category			
Disability mainly due to a non-psychiatric condition		6.45	7.199	0.723–57.518	*0.095*
Disability mainly due to a psychiatric condition		2.07	0.641	1.129–3.8	**0.019**

BD, bipolar disorder; *N*, number.

* In the past 2 years prior to study entry.The italic values indicate the presence of the trend (*p*<0.1).The bold values reflect the presence of statistical significance (*p*<0.05).

Table 5 shows the relations between an at least one physical comorbidity of BD and its associated factors (only the significant results and trends are listed). Key predictors comprised the presence of an at least one psychiatric comorbidity, various psychoactive substance-related disorders, female sex, age, age of onset, early onset of BD, duration of BD, atypical symptoms (particularly in females), a current episode of mild or moderate depression, unemployment, and pension (Table 5). Importantly, out of the psychiatric comorbidities, the presence of substance use disorders increased the odds of an at least one physical comorbidity of BD 2.67 times (95% CI: 1.097–6.483; *p* = 0.030). The subgroup analyses revealed the statistical significance of the substance use disorders to predict an at least one physical comorbidity in males (*N* = 76; OR 3.22; 95% CI: 1.031–10.073; *p* = 0.044). Interestingly, the current use of olanzapine in late-onset BD was associated with significantly decreased odds of an at least one physical comorbidity (*N* = 85; OR 0.29; 95% CI: 0.1–0.833; *p* = 0.022). Only trends were noted for associations between aripiprazole and an at least one physical comorbidities in all, in males, and in early-onset BD (Table 5). The current use of quetiapine in early-onset BD was associated with 2.42 times higher odds of an at least one physical comorbidity (*N* = 94; OR 2.42; 95% CI: 1.022–5.745; *p* = 0.044). Finally, no significant associations were found between the current use of valproate or lithium and at least one physical comorbidity.

**Table 5:** Factors associated with an at least one physical comorbidity of bipolar disorder (only the significant results and trends are listed).

	*N*	Odds ratio	Standard error	95% confidence interval	*p*-value
*Binary predictors*					
Male sex in all	179	0.42	0.135	0.221–0.786	**0.007**
Early onset of BD in all	179	1.04	0.015	1.01–1.069	**0.010**
Early onset of BD in females	103	1.04	0.021	0.996–1.078	*0.075*
Early onset of BD in males	76	1.05	0.025	0.997–1.096	*0.067*
Atypical symptoms in all	179	3.16	1.419	1.307–7.616	**0.011**
Atypical symptoms in females	103	5.43	4.217	1.187–24.869	**0.029**
Rapid cycling in late-onset BD	85	6.38	6.829	0.784–51.963	*0.083*
Use of olanzapine in all	179	0.49	0.19	0.229–1.049	*0.066*
Use of olanzapine in late-onset BD	85	0.29	0.156	0.1–0.833	**0.022**
Use of aripiprazole in all	179	0.35	0.195	0.114–1.046	*0.060*
Use of aripiprazole in males	76	0.19	0.162	0.037–1.00	*0.050*
Use of aripiprazole in early-onset BD	94	0.28	0.184	0.074–1.023	*0.054*
Use of quetiapine in early-onset BD	94	2.42	1.067	1.022–5.745	**0.044**
At least one psychiatric comorbidity in all	179	3.54	1.18	1.838–6.8	**< 0.001**
At least one psychiatric comorbidity in females	103	4.75	2.383	1.777–12.696	**0.002**
At least one psychiatric comorbidity in males	76	2.91	1.389	1.14–7.4167	**0.025**
At least one psychiatric comorbidity in early-onset BD	94	2.14	0.936	0.91–5.045	*0.081*
At least one psychiatric comorbidity in late-onset BD	85	7.02	3.807	2.423–20.322	**< 0.001**
Various psychoactive substance-related disorders	179	2.67	1.209	1.097–6.483	**0.030**
Borderline personality disorder (F60.3) in all	179	6.62	6.97	0.84–52.155	*0.073*
Specific personality disorders other than F60.3 in all	179	6.62	6.97	0.84–52.155	*0.073*
*Continuous predictors*					
Age in all	179	1.06	0.015	1.029–1.086	**< 0.001**
Age in females	103	1.05	0.019	1.013–1.086	**0.007**
Age in males	76	1.07	0.025	1.024–1.122	**0.003**
Age in early-onset BD	94	1.05	0.021	1.05–0.021	**0.017**
Age in late-onset BD	85	1.14	0.041	1.066–1.227	**< 0.001**
Age of onset in all	179	1.04	0.015	1.009–1.069	**0.010**
Age of onset in females	103	1.04	0.021	0.996–1.078	*0.075*
Age of onset in males	76	1.05	0.025	0.997–1.096	*0.067*
BD diagnosis duration in all	179	1.05	0.021	1.011–1.095	**0.012**
BD diagnosis duration in males	76	1.08	0.036	1.007–1.15	**0.029**
BD diagnosis duration in early-onset BD	94	1.06	0.025	1.008–1.105	**0.021**
Number of manic/hypomanic episodes in males*	39	6.45	7.07	0.751–55.335	*0.089*
*Factor variables*					
Pattern of BD illness progression in all:	179				
Chronic		A reference category			
Episodic with complete remissions		0.42	0.213	0.153–1.134	*0.087*
Episodic with incomplete remissions		0.59	0.32	0.204–1.708	0.331
Episodic with rapid cycling		0.82	0.767	0.13–5.138	0.830
*Current stage of BD in all*	177				
Currently in a remission		A reference category			
A current hypomanic episode		1.13	0.701	0.336–3.811	0.842
A current episode of mild or moderate depression		4.95	2.472	1.861–13.175	**0.001**
A current manic episode without psychotic symptoms		0.71	0.728	0.094–5.315	0.736
A current mixed episode		0.55	0.308	0.184–1.647	0.285
A current episode of severe depression without psychotic symptoms		2.12	1.329	0.622–7.241	0.230
A current manic episode with psychotic symptoms		0.35	0.442	0.031–4.087	0.405
A current episode of severe depression with psychotic symptoms		0.94	0.756	0.196–4.536	0.942
*Employment status*	179				
A full-time employment		A reference category			
A part-time employment		2.63	1.393	0.933–7.426	*0.068*
A casual employment		1.97	1.409	0.487–7.994	0.341
Not employed		3.22	1.302	1.461–7.113	**0.004**
Pensioners		12.72	8.631	3.364–48.09	**<0.001**

BD, bipolar disorder; *N*, number.

* In the past 2 years prior to study entry.The italic values indicate the presence of the trend (*p*<0.1).The bold values reflect the presence of statistical significance (*p*<0.05).

Table 6 reflects the phenotype of the study participants free of BD comorbidities (only the significant results are listed). Notably, an increased probability of the comorbidity-free BD was noted in those receiving olanzapine (observed in all, in males, and in late-onset BD), aripiprazole (observed in males and in early-onset BD), and in those with the episodic pattern of BD illness progression with complete remissions. The factors associated with the decreased odds of comorbidity-free BD included the unemployed status, pension, age and age of onset in late-onset BD, atypical symptoms (in all and in late-onset BD), and the use of quetiapine (in all, in males, and in early-onset BD) and valproate (in early-onset BD).

**Table 6:** Significant outcomes associated with the comorbidity-free bipolar disorder.

	*N*	Odds ratio	Standard error	95% confidence interval	*p*-value
*Binary predictors*					
Atypical symptoms in all	179	0.06	0.063	0.008–0.461	**0.007**
Atypical symptoms in late-onset BD	85	0.11	0.117	0.014–0.88	**0.037**
Use of olanzapine in all	179	2.7	1.116	1.206–6.071	**0.016**
Use of olanzapine in males	76	4.22	2.552	1.29–13.803	**0.017**
Use of olanzapine in late-onset BD	85	3.27	1.845	1.084–9.88	**0.035**
Use of aripiprazole in males	76	6.93	5.307	1.547–31.08	**0.011**
Use of aripiprazole in early-onset BD	94	3.78	2.529	1.017–14.028	**0.047**
Use of quetiapine in all	179	0.42	0.162	0.199–0.898	**0.025**
Use of quetiapine in males	76	0.26	0.151	0.084–0.811	**0.020**
Use of quetiapine in early-onset BD	94	0.16	0.098	0.049–0.53	**0.003**
Use of valproate in early-onset BD	94	0.28	0.161	0.093–0.86	**0.026**
*Continuous predictors*					
Age in late-onset BD	85	0.88	0.033	0.822–0.951	**0.001**
Age of onset in late-onset BD	85	0.89	0.039	0.814–0.968	**0.007**
*Factor variables*					
Pattern of BD illness progression in all:	179				
Chronic		A reference category			
Episodic with complete remissions		3.81	2.49	1.059–13.733	**0.041**
Episodic with incomplete remissions		1.36	0.98	0.332–5.582	0.669
Episodic with rapid cycling		2.78	2.83	0.376–20.5	0.316
Current stage of BD in all:	177				
Currently in a remission		A reference category			
A current hypomanic episode		0.40	0.322	0.081–1.943	0.254
A current episode of mild or moderate depression		0.10	0.073	0.021–0.426	**0.002**
A current manic episode without psychotic symptoms		2.18	2.253	0.288–16.511	0.450
A current mixed episode		0.99	0.593	0.307–3.2	0.989
A current episode of severe depression without psychotic symptoms		0.31	0.249	0.065–1.491	0.144
A current manic episode with psychotic symptoms		4.36	5.461	0.376–50.715	0.239
A current episode of severe depression with psychotic symptoms		1.64	1.319	0.337–7.942	0.541
*Employment status*	179				
A full-time employment		A reference category			
A part-time employment		0.4	0.224	0.133–1.199	0.102
A casual employment		0.8	0.570	0.198–3.236	0.754
Not employed		0.16	0.076	0.061–0.405	**< 0.001**
Pensioners		0.08	0.063	0.017–0.377	**0.001**

BD, bipolar disorder; N, number.The bold values reflect the presence of statistical significance (*p*<0.05).

Table 7 presents the relations between the psychoactive substance-related disorder (the most common psychiatric comorbidity of BD in our sample) and its associated factors. The results indicate that the odds of a substance use disorder in BD is 12 times higher given Attention deficit hyperactivity disorder (ADHD) comorbidity compared to no ADHD (Table 7). The presence of psychoactive substance-related disorders was significantly associated with an at least one physical comorbidity of BD, other specified diseases of the digestive system, disability mainly due to a psychiatric condition, and more frequent psychiatric follow-up appointments (Table 7).

**Table 7:** Factors associated with the psychoactive substance-related disorder in bipolar disorder.

In all (*N* = 179)	Odds ratio	Standard Error	95% Confidence Interval	*p*-value
*Binary predictors*				
Male sex	1.69	0.620	0.820–3.467	0.155
Early onset of BD	1.32	0.487	0.639–2.717	0.455
Combination treatment	0.73	0.320	0.308–1.722	0.471
Atypical symptoms	1.71	0.692	0.772–3.778	0.187
Seasonal pattern	1.04	0.386	0.504–2.151	0.914
Rapid cycling	0.55	0.358	0.154–1.968	0.358
High efficacy of psychopharmacotherapy	1.26	0.624	0.480–3.324	0.636
Organic non-psychotic disorders	0.92	0.751	0.188–4.541	0.922
Psychotic disorders from the schizophrenia spectrum	1.24	1.454	0.126–12.302	0.852
Panic disorder	1	omitted	-	-
Generalized anxiety disorder	1	omitted	-	-
Adjustment disorder with anxiety	0.32	0.339	0.04–2.555	0.282
Post-traumatic stress disorder	0.93	1.0499	0.1–8.533	0.946
Eating disorders	1.9	1.686	0.335–10.804	0.468
Sleep disorders	0.35	0.377	0.044–2.856	0.330
Sexual dysfunction	1	omitted	-	-
Borderline personality disorder (F60.3)	2.52	1.517	0.774–8.202	0.125
Specific personality disorders other than F60.3	1.12	0.769	0.293–4.301	0.866
Attention-deficit hyperactivity disorders	12	14.041	1.211–118.889	**0.034**
Other psychiatric disorders	1	omitted	-	-
At least one physical comorbidity	2.67	1.209	1.097–6.483	**0.030**
Essential (primary) hypertension	1.21	0.459	0.571–2.543	0.625
Obesity	1.45	0.704	0.56–3.757	0.444
Disorders of lipoprotein metabolism and other lipidemias	1.54	0.754	0.592–4.021	0.375
Chronic ischemic heart disease	0.63	0.409	0.173–2.256	0.473
Various other diseases of the musculoskeletal system and connective tissue	1.49	0.837	0.497–4.482	0.476
Headache	1.39	0.855	0.417–4.64	0.592
Disorders of thyroid gland	1.01	0.687	0.268–3.83	0.985
Various other neurological diseases	2.25	1.476	0.623–8.139	0.216
Other specified diseases of the digestive system	7.73	5.086	2.131–28.063	**0.002**
Type 2 diabetes mellitus	0.4	0.424	0.049–3.23	0.387
*Continuous predictors*				
Age	1.0	0.014	0.973–1.026	0.959
Age of onset	0.99	0.015	0.965–1.025	0.719
BD diagnosis duration	1.01	0.019	0.971–1.045	0.704
Number of depressive episodes*	0.83	0.136	0.604 - 1.148	0.264
Number of manic/hypomanic episodes*	0.60	0.237	0.276 - 1.3	0.195
Number of mixed episodes*	0.81	0.368	0.328 - 1.973	0.635
*Factor variables*				
Pattern of BD illness progression:				
Chronic	A reference category			
Episodic with complete remissions	0.90	0.483	0.317–2.574	0.849
Episodic with incomplete remissions	1.08	0.605	0.363–3.236	0.886
Episodic with rapid cycling	1.22	1.146	0.195–7.675	0.830
*Current stage of BD*				
Currently in a remission	A reference category			
A current hypomanic episode	1.95	1.315	0.519–7.316	0.323
A current episode of mild or moderate depression	1.46	0.663	0.601–3.554	0.403
A current manic episode without psychotic symptoms	1.46	1.746	0.141–15.203	0.751
A current mixed episode	1.01	0.719	0.251–4.072	0.987
A current episode of severe depression without psychotic symptoms	1.01	0.719	0.251–4.072	0.987
A current manic episode with psychotic symptoms	2.19	2.768	0.185–26.046	0.534
A current episode of severe depression with psychotic symptoms	0.73	0.821	0.081–6.602	0.780
*Interval of psychiatric follow-up appointments*				
Weekly	A reference category			
Monthly	0.42	0.213	0.155–1.134	*0.087*
Quarterly	0.18	0.123	0.045–0.694	**0.013**
Yearly	1	(empty)	-	-
*Employment status*				
A full-time employment	A reference category			
A part-time employment	0.48	0.406	0.092–2.521	0.386
A casual employment	1.32	1.178	0.230–7.585	0.755
Not employed	2.47	1.204	0.954–6.419	*0.063*
Pensioners	0.98	0.624	0.280–3.417	0.973
*Permanent disability*				
No disability	A reference category			
Disability mainly due to a non-psychiatric condition	3.46	3.193	0.565–21.137	0.180
Disability mainly due to a psychiatric condition	2.83	1.134	1.291–6.209	**0.009**

BD, bipolar disorder; *N*, number.

* In the past 2 years prior to study entry.The bold values reflect the presence of statistical significance (*p*<0.05).

**Figure 7:**
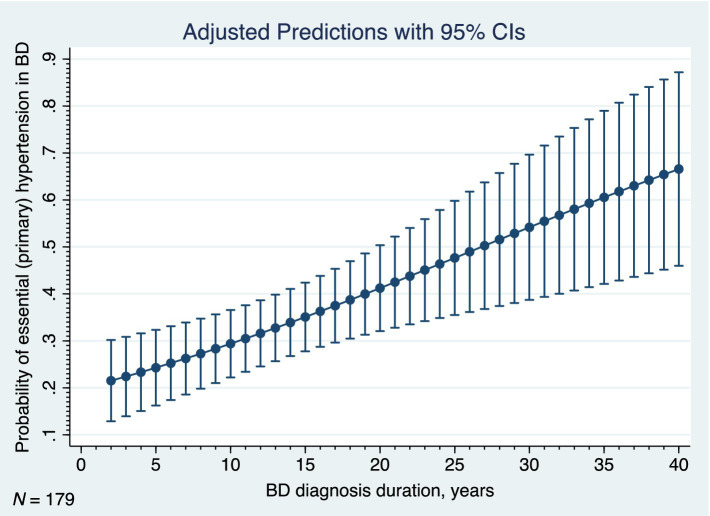
Adjusted predictions of duration of bipolar disorder for probability of hypertension, BD, bipolar disorder, 95% CI, 95% confidence interval, *N*, number.

Finally, we examined the relationships between the essential (primary) hypertension (the most common physical comorbidity of BD in our sample) and its correlates. The results of the logistic regression analyses are listed in Table 8. As for the clinical phenotype of BD, the age, age of onset, duration of BD, and the current episode of mild or moderate depression each increased occurrence of the hypertension (Table 8). The early onset of BD was associated with significantly decreased odds of the hypertension (OR 0.34; 95% CI: 0.178–0.648; *p* = 0.001). Out of the BD comorbidities, the adjustment disorder with anxiety, obesity, hyperlipidemia, ischemic heart disease, other specified diseases of the digestive system, and various other diseases of the musculoskeletal system and connective tissue were significantly associated with the hypertension in our study (Table 8). Figure 7 shows the adjusted predictions of BD duration for probability of the essential (primary) hypertension. The graph displays the computed predicted probability of the hypertension with its 95% CI for the BD diagnosis durations ranging from two to 40 years. The findings suggest that each 5 years of BD diagnosis duration are associated with an approximately 5–7% increase in the probability of the hypertension.

**Table 8:** Factors associated with the essential (primary) hypertension in bipolar disorder.

In all (*N* = 179)	Odds ratio	Standard error	95% confidence interval	*p*-value
*Binary predictors*				
Male sex	0.63	0.205	0.33–1.19	0.153
Early onset of BD	0.34	0.112	0.178–0.648	**0.001**
Combination treatment	0.82	0.322	0.38–1.769	0.613
Atypical symptoms	1.3	0.477	0.634–2.669	0.473
Seasonal pattern	1.13	0.363	0.603–2.12	0.701
Rapid cycling	1.15	0.548	0.455–2.925	0.763
High efficacy of psychopharmacotherapy	0.86	0.345	0.39–1.887	0.702
Organic non-psychotic disorders	1.35	0.896	0.365–4.961	0.656
Various psychoactive substance-related disorders	1.21	0.459	0.571–2.543	0.625
Psychotic disorders from the schizophrenia spectrum	0.66	0.764	0.067–6.438	0.717
Panic disorder	0.99	1.225	0.088–11.159	0.995
Generalized anxiety disorder	6.21	7.241	0.632–61.032	0.117
Adjustment disorder with anxiety	6.82	4.691	1.773–26.256	**0.005**
Post-traumatic stress disorder	0.49	0.55	0.0533–4.458	0.524
Eating disorders	0.99	0.873	0.176–5.572	0.992
Sleep disorders	2.53	1.59	0.74–8.669	0.139
Sexual dysfunction	6.21	7.241	0.632–61.032	0.117
Borderline personality disorder (F60.3)	1.78	1.032	0.57–5.546	0.322
Specific personality disorders other than F60.3	0.57	0.389	0.152–2.168	0.413
Attention-deficit hyperactivity disorders	1	(omitted)	-	**-**
Other psychiatric disorders	2.04	1.694	0.398–10.401	0.393
At least one physical comorbidity	1	(omitted)	-	**-**
Obesity	3.27	1.423	1.396–7.674	**0.006**
Disorders of lipoprotein metabolism and other lipidemias	8.73	4.386	3.259–23.37	**< 0.001**
Chronic ischemic heart disease	15.29	9.954	4.266–54.777	**< 0.001**
Various other diseases of the musculoskeletal system and connective tissue	2.78	1.399	1.033–7.452	**0.043**
Headache	1.83	0.997	0.631–5.321	0.265
Disorders of thyroid gland	1.54	0.871	0.509–4.666	0.444
Various other neurological diseases	1.71	1.074	0.501–5.856	0.391
Other specified diseases of the digestive system	3.8	2.462	1.066–13.533	**0.040**
Type 2 diabetes mellitus	5.11	3.624	1.271–20.524	0.022
*Continuous predictors*				
Age	1.11	0.019	1.069–1.143	**< 0.001**
Age of onset	1.07	0.016	1.039–1.102	**< 0.001**
BD diagnosis duration	1.05	0.018	1.019–1.089	**0.002**
Number of depressive episodes*	0.97	0.114	0.774–1.224	0.817
Number of manic/hypomanic episodes*	1.17	0.234	0.792–1.735	0.427
Number of mixed episodes*	1.03	0.335	0.546–1.948	0.924
*Factor variables*				
Pattern of BD illness progression:				
Chronic	A reference category			
Episodic with complete remissions	0.48	0.214	0.203–1.149	0.100
Episodic with incomplete remissions	0.46	0.219	0.183–1.167	0.102
Episodic with rapid cycling	1	(empty)	-	-
*Current stage of BD*				
Currently in a remission	A reference category			
A current hypomanic episode	1.19	0.785	0.328–4.335	0.789
A current episode of mild or moderate depression	2.27	0.898	1.047–4.928	**0.038**
A current manic episode without psychotic symptoms	0.9	1.061	0.088–9.138	0.925
A current mixed episode	0.38	0.308	0.08–1.848	0.232
A current episode of severe depression without psychotic symptoms	2.09	1.192	0.682–6.395	0.197
A current manic episode with psychotic symptoms	1.34	1.683	0.115–15.671	0.814
A current episode of severe depression with psychotic symptoms	2.01	1.63	0.412–9.842	0.387
*Interval of psychiatric follow-up appointments*				
Weekly	A reference category			
Monthly	2.26	1.337	0.711–7.203	0.167
Quarterly	1.85	1.217	0.507–6.718	0.352
Yearly	4	6.083	0.203–78.791	0.362
*Employment status*				
A full-time employment	A reference category			
A part-time employment	2.7	1.552	0.875–8.332	*0.084*
A casual employment	1.13	0.992	0.2–6.336	0.894
Not employed	1.97	0.926	0.783–4.949	0.150
Pensioners	7.5	4.012	2.629–21.398	**< 0.001**
*Permanent disability*				
No disability	A reference category			
Disability mainly due to a non-psychiatric condition	3.8	3.401	0.658–21.955	0.136
Disability mainly due to a psychiatric condition	0.82	0.268	0.435–1.559	0.550

BD, bipolar disorder; *N*, number.

* In the past 2 years prior to study entry.The bold values reflect the presence of statistical significance (*p*<0.05).

### 3.3. Multivariate analyses

Multiple regression models were created to predict: (i) at least one psychiatric comorbidity of BD; (ii) at least one physical comorbidity of BD; (iii) the psychoactive substance-related disorder; (iv) the essential (primary) hypertension; (v) cardiovascular comorbidities of BD; (vi) endocrine, nutritional and metabolic comorbidities of BD; and (vii) clinical phenotype of comorbidity-free BD.

(i) In a model adjusting for sex, early onset of BD, BD diagnosis duration and pattern of BD illness progression, the presence of an at least one physical comorbidity (OR 2.89; 95% CI: 1.336–6.243; *p* = 0.007), the atypical symptoms of BD (OR: 3.35; 95% CI: 1.403–7.996; *p* = 0.006), and the unemployed status (OR 3.73; 95% CI: 1.447–9.62; *p* = 0.006) were each independently associated with an at least one psychiatric comorbidity in the entire sample (*p* < 0.001).
(ii) In a model adjusting for use of antipsychotics, pattern of BD illness progression, and atypical symptoms of BD, the presence of an at least one psychiatric comorbidity (OR 2.64; 95% CI: 1.215–5.722; *p* = 0.014), sex (for male sex: OR 0.44; 95% CI: 0.21–0.912; *p* = 0.027), BD diagnosis duration (OR 1.05; 95% CI: 1.002–1.102; *p* = 0.040) and pension (OR 7.53; 95% CI: 1.796–31.554; *p* = 0.006) were each independently associated with an at least one physical comorbidity in the entire sample (*p* < 0.001). When “at least one psychiatric comorbidity” was replaced by “various psychoactive substance-related disorders” in this regression model, the sex, BD diagnosis duration and pension maintained significant independent associations with an at least one physical comorbidity in the entire sample (*p* < 0.001). In addition, the various psychoactive substance-related disorders (OR 2.77; 95% CI: 1.024–7.482; *p* = 0.045), atypical symptoms of BD (OR 2.74; 95% CI: 1.029–7.306; *p* = 0.044), unemployed status (OR 2.72; 95% CI: 1.054–7.04; *p* = 0.039) and use of antipsychotics (OR 0.47; 95% CI: 0.225–0.99; *p* = 0.047) were each independently associated with an at least one physical comorbidity in the entire sample (*p* < 0.001).
(iii) In a model adjusting for BD diagnosis duration and pattern of BD illness progression, the male sex (OR 2.22; 95% CI: 1.004–4.919; *p* = 0.049), presence of an at least one physical comorbidity (OR 3.2; 95% CI: 1.206–8.469; *p* = 0.019), disability mainly due to a psychiatric condition (OR 2.75; 95% CI: 1.162–6.51; *p* = 0.021) and ADHD (OR 14.83; 95% CI: 1.034–212.759; *p* = 0.047) were each independently associated with a psychoactive substance-related disorder in the entire sample (*p* = 0.011). When “at least one physical comorbidity” was replaced by “other specified diseases of the digestive system” in this regression model, the disability mainly due to a psychiatric condition and ADHD maintained significant independent associations with a psychoactive substance-related disorder in the entire sample (*p* < 0.001), and the “other specified diseases of the digestive system” predicted the psychoactive substance-related disorder independent of sex (OR 13.84; 95% CI: 3.185–60.093; *p* < 0.001).
(iv) In a model adjusting for sex, obesity and pattern of BD illness progression, the BD diagnosis duration (OR 1.06; 95% CI: 1.022–1.098; *p* = 0.002), adjustment disorder with anxiety (OR 5.76; 95% CI: 1.309–25.326; *p* = 0.021) and use of olanzapine (OR 0.34; 95% CI: 0.122–0.963; *p* = 0.042) were each independently associated with an essential (primary) hypertension in the entire sample (*p* < 0.001).
(v) In a model adjusting for sex, pattern of BD illness progression and use of antipsychotics, the BD diagnosis duration (OR 1.07; 95% CI: 1.028–1.106; *p* = 0.001), adjustment disorder with anxiety (OR 5.57; 95% CI: 1.29–24.068; *p* = 0.021) and obesity (OR 3.11; 95% CI: 1.187–8.162; *p* = 0.021) were each independently associated with an at least one cardiovascular comorbidity of BD in the entire sample (*p* < 0.001). Similar associations were observed when “antipsychotics” were replaced by “olanzapine,” “aripiprazole” or “quetiapine.” Significant associations persisted when “obesity” was replaced by “hyperlipidemia” (OR 7.55; 95% CI: 2.549–22.349; *p* < 0.001) or “at least one endocrine/nutritional/metabolic disease” (OR 2.76; 95% CI: 1.357–5.597; *p* = 0.005) in this multiple logistic regression model.
(vi) In a model adjusting for sex, rapid cycling and use of antipsychotics, both the BD diagnosis duration (OR 1.04; 95% CI: 1.006–1.076; *p* = 0.021) and the adjustment disorder with anxiety (OR 3.62; 95% CI: 1.034–12.654; *p* = 0.044) were each independently associated with an at least one endocrine, nutritional or metabolic comorbidity of BD in the entire sample (*p* = 0.019).
(vii) In a model adjusting for sex, BD diagnosis duration, and pattern of BD illness progression, the atypical symptoms (OR 0.05; 95% CI: 0.006–0.423; *p* = 0.006), the unemployed status (OR 0.13; 95% CI: 0.04–0.393; *p* < 0.001), the pension (OR 0.07; 95% CI: 0.012–0.41; *p* = 0.003), and the use of antipsychotics (OR 3.05; 95% CI: 1.247–7.47; *p* = 0.015) were each independently associated with a comorbidity-free BD in the entire sample (*p* < 0.001). Similar associations were observed when “antipsychotics” were replaced by “olanzapine.”

## 4. Discussion

The primary findings of the present cross-national study are the novel estimates and clinical correlates related to both the comorbidity phenotype and the comorbidity-free phenotype of adults with BD in Slovakia. This study has shown that out of 179 study participants, 22.4% were free of comorbidity, 42.5% had both psychiatric and physical comorbidities, 53.6% had at least one psychiatric comorbidity, and 66.5% had at least one physical comorbidity. The 10 most prevalent comorbid conditions were the essential hypertension (33.5%), various psychoactive substance-related disorders (21.2%), specific personality disorders (14.6%), in particular, the borderline personality disorder (7.3%), obesity (14.5%), lipid disorders (14%), ischemic heart disease (11.2%), various other diseases of the musculoskeletal system and connective tissue (10.1%), headache (8.4%), disorders of thyroid gland (7.8%), and the adjustment disorder with anxiety (6.7%). These findings are partially in line with previous research (10, 43, 44) reporting a high prevalence of comorbid physical conditions in BD. Compared to a large national cohort study of 6,618 Swedish adults with BD (10), the current study has demonstrated similar rates of the various psychoactive substance-related disorders, higher rates of an at least one physical comorbidity, cardiovascular disease, hypertension, ischemic heart disease and lipid disorders, and lower rates of the diabetes mellitus and cancer. Our results also share many similarities with Kilbourne et al.’s findings (43) and Soreca et al.’s findings (44). The evidence we found points to confirming that a substantial burden of general medical conditions is related to duration of bipolar illness. Specifically, the high rates of hypertension, hyperlipidemia, obesity, thyroid dysfunction, and psychoactive substance-related disorders observed in our study substantiate previous findings in the literature (10, 43–45).

With respect to physical comorbidities in our study it is fundamental to note that the highest rates were observed for the individual components of the metabolic syndrome (hypertension, obesity, lipid disorders, and type 2 diabetes mellitus). While the prevalence of type 2 diabetes in our bipolar sample 5.6% was considerably lower than the prevalence estimates 9.4% (19) and 11% (20) in the previous meta-analyses, the median age of our study participants was 49 years. Importantly, although type 2 diabetes is most often diagnosed between the ages 45 and 64, the highest rates of diabetes are among ages 65 years and older. Remarkably, age-stratified analyses of our data revealed the following increasing rates of type 2 diabetes: 5.7% in those older than 20 years, 6.1% in those older than 30 years, 7.3% in those older than 40 years, 8.9% in those older than 50 years, and 13.5% in those older than 60 years. Despite multicollinearity, both physiological aging and longer duration of BD seem to be prominent risk factors for type 2 diabetes in persons with BD. Additional stratified analyses have proved that the rates of type 2 diabetes in our study population increase with BD duration: 6.8% in those with BD duration more than 10 years, 11.5% in those with BD duration more than 15 years, and 19.4% in those with BD duration more than 20 years. These values correlate favorably with previous heterogenous findings in the literature and further support the idea that BD might share some of the common risk factors for the development of prediabetes and its progression to overt type 2 diabetes (19, 20). The observed interaction merits a regular comprehensive screening for type 2 diabetes, particularly in persons older than 50 years with BD of more than 15 years duration. Early diagnosis would thus allow for a timely initiation of the complex targeted treatment in this at-risk population. Future research should investigate the extent to which novel prevention initiatives and strategies can prevent or delay the onset of type 2 diabetes in BD. As regards obesity and hyperlipidemia, both may potentially reflect different etiologies, including but not limited to genetic factors and epigenetic behavioral and treatment-related side effects, as well as to altered levels of appetite-regulating hormones in BD (21). Evidence from systematic review suggests that dyslipidemia and insulin resistance have shown a bidirectional relationship with the prevalence and severity of BD (22). Given the cardiometabolic sequelae of BD, it is important for providers to be aware of their patient’s metabolic risk factors in order to customize pharmacotherapy and make the proactive best-informed decisions in plan of care.

Interestingly, a significantly higher prevalence of the cardiovascular (hypertension, ischemic heart disease, peripheral vascular disease) but not metabolic comorbidities was observed in our study participants with late-onset BD. The reasons for this result are not yet entirely understood. It cannot be ignored that while both the metabolic and the cardiovascular diseases are age-related, the subjects with late-onset BD were older and had a shorter duration of BD compared to the early-onset subgroup. The interaction effect between the older age and the possibly delayed treatment of BD in the late-onset subgroup could have contributed to a higher rate of cardiovascular diseases in this subgroup. Within the context of our results it can be conceivably hypothesized that specifically the higher prevalence of hypertension in late-onset BD might be partially related to delayed guideline-recommended psychopharmacological interventions. It has been demonstrated that both (hypo) mania and essential hypertension share biological background with a number of similarities in their genetics, underlying personality and temperamental factors, precipitating factors, comorbidity and response to treatment (23). As a result, the hypertension might be one of the faces of bipolar (hypo) mania, and (hypo) mania can be compared to the hypertension of the mood (45). On the other hand, the longer duration of BD and associated treatment-related side effects in the early-onset subgroup may have accelerated the development and progression of metabolic sequelae at a rapid rate. Therefore, the accumulated metabolic comorbidities might occur at an earlier age than in the late-onset subgroup. It can thus be reasonably assumed that compared to early-onset BD, persons with late-onset BD reported higher rates of the cardiovascular but not metabolic comorbidities. In multiple regression models, the use of olanzapine was associated with independently decreased odds of the hypertension and was linked with the clinical phenotype of comorbidity-free BD. Because of the cross-sectional design of the study, no conclusions on causality or directions of the associations can be drawn, and noncausal explanations are possible. The observed findings might be explained by the fact that the long-term olanzapine treatment is not recommended for persons with diabetes or metabolic syndrome/its component factors (including hypertension). Another explanation based on the results of the present study is that olanzapine was associated with complete remissions during the episodic course of BD illness progression in adults free of BD comorbidities. Importantly, the results of the large Finnish nationwide study (24) demonstrated that current antipsychotic use was associated with greater adherence to cardiometabolic medications, which might explain reduced cardiovascular mortality observed with antipsychotics in people with schizophrenia. The findings implicate that timely diagnosis and efficacious psychopharmacological treatment are critical to reduce the cardiovascular disease risk in adults with BD.

Finally, high rates of BD comorbid diseases of the musculoskeletal system and connective tissue, thyroid gland disorders, and headache reported in our study substantiate previous findings in the literature (9, 25, 43). Besides the well-known suppressive effects of lithium on the thyroid (26), evidence suggests that genetically predicted higher level of free thyroxine is significantly associated with a reduced risk of BD (46). In addition, a recent study suggests that novel circadian rhythm disruptions may link BD with endocrine comorbidities (47). Future studies should concentrate on the investigation of the therapeutic potential for novel interventions targeting circadian rhythms and on identification of other potential therapeutic targets.

As regards the psychiatric comorbidity, our results share a number of similarities with previous findings (8, 10). The most prevalent various psychoactive substance-related disorders were strongly associated with the presence of atypical symptoms of BD (particularly in females), rapid cycling in females, seasonal pattern in late-onset BD, and with the longer duration of BD in the late-onset subgroup. In addition, strong associations of the substance use disorder with an at least one physical comorbidity (particularly with hypertension, obesity, hyperlipidemia, headache), disability mainly due to a psychiatric condition, pension and unemployed status were proved. The remarkable results to emerge from the data are the observed positive associations of the substance use disorder both with a current hypomanic episode and a current episode of mild or moderate depression and the independent associations with ADHD. This is in good agreement with the results of the recent meta-analysis (48), suggesting that comorbid BD and ADHD may have some distinctive clinical features including an earlier onset of BD and higher comorbid alcohol/substance use disorder rates (48). Meta-analytic results further suggest that an early (compared to late) age of onset in BD is associated with a longer delay to treatment, greater severity of depression, and with higher levels of comorbid anxiety and substance abuse (49, 50).

Data about the prevalence of borderline personality disorder and BD comorbidity are scarce (27, 28, 51). A systematic review and meta-analysis from 2016 has demonstrated the prevalence of comorbid borderline personality disorder among 5,273 people with BD was 21.6% (95% CI: 17.0–27.1), and the prevalence of BD among 1814 people with borderline personality disorder was 18.5% (95% CI: 12.7–26.1) (27). In our study, 7.3% of participants experienced comorbid borderline personality disorder. Even though these findings differ from previous results reported in the literature, this information is important to develop appropriate treatments for subjects with both disorders, improve their clinical presentation/course, and prevent the increased risk of suicidality. Further research is required to explore the reasons for the variability observed in the prevalence estimates.

The adjustment disorder with anxiety was reported in 6.7% of our study participants. This psychiatric comorbidity was strongly associated with the late onset of BD, hypertension, and cardiometabolic comorbidities of BD. The observations from our study are consistent with previous evidence suggesting that anxiety symptoms are highly prevalent among individuals with BD (29–31). Available evidence indicates a three-fold increase in the prevalence of anxiety disorders in those with BD (29). Importantly, BD-anxiety comorbidity may be associated with an unfavorable clinical profile leading to poorer outcomes (e.g., an increased number of suicide attempts, rapid cycling, lifetime alcohol abuse, psychosis) (30).

Finally, sleep disorders and eating pathology may occur comorbidly with BD due to well-established shared underlying pathophysiological features. More comprehensive investigations in this field would be of great help to guide novel psychopharmacological and psychotherapeutic interventions to reestablish the functionality of dysregulated emotion and impulsivity in the development of sleep disorders and eating pathology in BD.

Growing evidence points to similar neuropsychological signatures and the involvement of shared biological pathways between BD and its comorbidity. Several possible pathophysiological mechanisms have been proposed to link BD with cardio-metabolic comorbidities, including but not limited to hypothalamic–pituitary–adrenal axis dysfunction (32), thyroid hormone abnormalities (33), mitochondrial dysfunction (34), chronic inflammatory state related immune dysfunctions (35, 36), impaired fatty acid and phospholipid metabolism (37), purinergic system dysfunction (38), dysregulation of glycogen synthase kinase-3ß (GSK-3ß) (39), and dysregulation of noradrenaline signaling (40). While there are many important neurobiological and pathophysiological underpinnings of BD and its comorbidity, further research is required to fully explore the mechanisms and translate the findings into clinical practice.

One of the strengths of the present nationwide multicenter study was the relatively large sample size of 179 participants randomly selected from outpatient psychiatric facilities across Slovakia. The availability of outpatient diagnoses is an important strength because it allows the inclusion of milder, nonhospitalized comorbidities and enables more generalizable risk estimates. This avoids bias that may potentially result from the sole use of inpatient data and improves the generalizability of the results to the target population. Another strength of the current study is that it represents a comprehensive examination of the adult population with BD based on the in-depth analysis of the selected demographic and clinical variables controlling for key covariates in analyses. Limitations include the cross-sectional design of the study, which precludes making inferences of causality and evaluating the outcomes longitudinally. The study did not report the number of persons with BD subtypes that could enable to further investigate differences between type I and type II of BD. Specific underlying mechanisms of BD comorbidities remain not completely understood and warrant additional studies using longitudinal sociodemographic and clinical data. The findings should be interpreted with caution also because of possible confounding by disease severity or other unmeasured factors. Individual data about smoking, physical activity, or other direct lifestyle measurements were unavailable. Also, detailed information about the types of various psychoactive substance use disorders (alcohol, illegal drugs, and medications) was unavailable.

To summarize, we have reported novel estimates and clinical correlates related to both the comorbidity-free phenotype and to the factors associated with psychiatric and physical comorbidities in adults with BD in Slovakia. In the current study, BD was more strongly associated with an at least one psychiatric comorbidity among unemployed adults with atypical symptoms of BD and with an at least one physical comorbidity. Further, BD was more strongly associated with an at least one physical comorbidity among female pensioners with an at least one psychiatric comorbidity (in particular, the substance use disorder) and with a longer duration of BD. Notably, BD was more strongly associated with a substance use disorder among males disabled mainly due to a psychiatric condition with an at least one physical comorbidity and ADHD. The most common physical comorbidity, the hypertension, was typically reported by study participants with a longer duration of BD suffering from a comorbid adjustment disorder with anxiety who were currently not treated with olanzapine. Finally, while the comorbidity-free BD was strongly associated with the current use of olanzapine, it was least strongly associated with atypical symptoms of BD among those who were unemployed or pensioners. Our results supplement and extend existing data describing the clinical overlap and interactions between BD and psychiatric and physical comorbidity. The findings provide new insights into understanding of the clinical presentation and course of BD that can inform clinical practice and further research to continue to investigate potential mechanisms of BD adverse outcomes and disease complications onset. Evidence suggests the comorbid disorders in BD are associated with several indices of harmful dysfunction, decrements in functional outcomes, increased utilization of medical services (11), and can negatively affect the course of BD, severity of BD and its treatment (44). Ultimately, BD comorbidities can lead to even greater disability and mortality (41). Our findings indicate that the associations between psychiatric and somatic comorbidities of BD seem to be bidirectional. This is consistent with previous studies that have supported for shared pathogenic mechanisms in mental disorders and somatic diseases (52–54). Novel prevention initiatives and effective strategies need to be identified and successfully implemented in clinical settings to prevent, slow down or delay the onset of comorbid pathologies seen in BD.

## Data availability statement

The raw data supporting the conclusions of this article will be made available by the authors, without undue reservation.

## Ethics statement

The studies involving human participants were reviewed and approved by the Ethics Committee of the Kosice Self-governing Region (study protocol number: 4670/2015/OSVaZ-28576). The patients/participants provided their written informed consent to participate in this study.

## Author contributions

JD, PV, and MP: conceptualization. JD and MP: methodology, writing — original draft preparation, and visualization. MP: software. MM and MP: formal analysis. JD, MM, PV, and MP: investigation, writing — review and editing. JD, MM, and PV: resources. JD and MM: data curation. JD: supervision, project administration, and funding acquisition. All authors contributed to the article and approved the submitted version.

## Conflict of interest

JD and MM received honoraria or consultation fees outside of this work from KRKA Slovakia.

The remaining authors declare that the research was conducted in the absence of any commercial or financial relationships that could be construed as a potential conflict of interest. The authors declare that this study received funding from KRKA Slovakia. The funder had the following involvement in the study: support to data collection.

## Publisher’s note

All claims expressed in this article are solely those of the authors and do not necessarily represent those of their affiliated organizations, or those of the publisher, the editors and the reviewers. Any product that may be evaluated in this article, or claim that may be made by its manufacturer, is not guaranteed or endorsed by the publisher.

## References

[1] Grande I Berk M Birmaher B Vieta E. Bipolar disorder. Lancet. (2016) 387:1561–72. doi: 10.1016/S0140-6736(15)00241-X 26388529

[2] American Psychiatric Association (2022). Diagnostic and statistical manual of mental disorders, Fifth Edition, Text Revision (DSM-5-TR). Washington, D.C.

[3] Clemente AS Diniz BS Nicolato R Kapczinski FP Soares JC Firmo JO. Bipolar disorder prevalence: a systematic review and meta-analysis of the literature. Rev Brasileira Psiquiatria. (1999) 37:155–61. 10.1590/1516-4446-2012-1693 25946396

[4] Global Burden of Disease Study (2019). Bipolar disorder prevalence, males vs. females, 2019 Seattle, United States: Institute for Health Metrics and Evaluation (IHME); 2020 [estimated share of males versus females who have bipolar disorder, whether or not they are diagnosed, based on representative surveys, medical data and statistical modelling.]. Available from: https://vizhub.healthdata.org/gbd-results/ (Accessed June 22, 2023).

[5] (NIMH) NIoMH (2023) Bipolar Disorder. Available at: https://www.nimh.nih.gov/health/statistics/bipolar-disorder (Accessed March 05, 2023).

[6] Bolton S Warner J Harriss E Geddes J Saunders KEA. Bipolar disorder: Trimodal age-at-onset distribution. Bipolar Disord. (2021) 23:341–56. doi: 10.1111/bdi.13016, PMID: 33030292 PMC8359178

[7] Scott J Graham A Yung A Morgan C Bellivier F Etain B. A systematic review and meta-analysis of delayed help-seeking, delayed diagnosis and duration of untreated illness in bipolar disorders. Acta Psychiatr Scand. (2022) 146:389–405. doi: 10.1111/acps.13490, PMID: 36018259

[8] McElroy SL Altshuler LL Suppes T Keck PE Jr Frye MA Denicoff KD. Axis I psychiatric comorbidity and its relationship to historical illness variables in 288 patients with bipolar disorder. Am J Psychiatry. (2001) 158:420–6. doi: 10.1176/appi.ajp.158.3.420, PMID: 11229983

[9] Pahwa M Kucuker MU Ho MC Puspitasari A Moore KM Betcher HK. Cardiometabolic and endocrine comorbidities in women with bipolar disorder: a systematic review. J Affect Disord. (2023) 323:841–59. doi: 10.1016/j.jad.2022.12.023, PMID: 36538952

[10] Crump C Sundquist K Winkleby MA Sundquist J. Comorbidities and mortality in bipolar disorder: a Swedish national cohort study. JAMA Psychiatry. (2013) 70:931–9. doi: 10.1001/jamapsychiatry.2013.1394, PMID: 23863861

[11] McIntyre RS Konarski JZ Soczynska JK Wilkins K Panjwani G Bouffard B. Medical comorbidity in bipolar disorder: implications for functional outcomes and health service utilization. Psychiatric Ser. (2006) 57:1140–4. doi: 10.1176/ps.2006.57.8.1140, PMID: 16870965

[12] Warner A Holland C Lobban F Tyler E Harvey D Newens C. Physical health comorbidities in older adults with bipolar disorder: a systematic review. J Affect Disord. (2023) 326:232–42. doi: 10.1016/j.jad.2023.01.083, PMID: 36709829

[13] Wiström ED O'Connell KS Karadag N Bahrami S Hindley GFL Lin A. Genome-wide analysis reveals genetic overlap between alcohol use behaviours, schizophrenia and bipolar disorder and identifies novel shared risk loci. Addiction. (2022) 117:600–10. doi: 10.1111/add.15680, PMID: 34472679

[14] Bharadhwaj VS Mubeen S Sargsyan A Jose GM Geissler S Hofmann-Apitius M. Integrative analysis to identify shared mechanisms between schizophrenia and bipolar disorder and their comorbidities. Prog Neuro-Psychopharmacol Biol Psychiatry. (2023) 122:110688. doi: 10.1016/j.pnpbp.2022.110688, PMID: 36462601

[15] Altaf-Ul-Amin M Hirose K Nani JV Porta LC Tasic L Hossain SF. A system biology approach based on metabolic biomarkers and protein-protein interactions for identifying pathways underlying schizophrenia and bipolar disorder. Sci Rep. (2021) 11:14450. doi: 10.1038/s41598-021-93653-3, PMID: 34262063 PMC8280132

[16] Vandenbroucke JP von Elm E Altman DG Gøtzsche PC Mulrow CD Pocock SJ. Strengthening the reporting of observational studies in epidemiology (STROBE): explanation and elaboration. PLoS Med. (2007) 4:e297. doi: 10.1371/journal.pmed.0040297, PMID: 17941715 PMC2020496

[17] WHO (2016). International statistical classification of diseases and related health problems 10th revision ICD-10 version: 2016. Available at: https://icd.who.int/browse10/2016/en (Accessed March 06, 2023).

[18] VanderWeele TJ Shpitser I. A new criterion for confounder selection. Biometrics. (2011) 67:1406–13. doi: 10.1111/j.1541-0420.2011.01619.x, PMID: 21627630 PMC3166439

[19] Vancampfort D Mitchell AJ De Hert M Sienaert P Probst M Buys R. Prevalence and predictors of type 2 diabetes mellitus in people with bipolar disorder: a systematic review and meta-analysis. J Clin Psychiatry. (2015) 76:1490–9. doi: 10.4088/JCP.14r09635, PMID: 26214054

[20] Lindekilde N Scheuer SH Rutters F Knudsen L Lasgaard M Rubin KH. Prevalence of type 2 diabetes in psychiatric disorders: an umbrella review with meta-analysis of 245 observational studies from 32 systematic reviews. Diabetologia. (2022) 65:440–56. doi: 10.1007/s00125-021-05609-x, PMID: 34841451

[21] Misiak B Kowalski K Stańczykiewicz B Bartoli F Carrà G Samochowiec J. Appetite-regulating hormones in bipolar disorder: a systematic review and meta-analysis. Front Neuroendocrinol. (2022) 67:101013. doi: 10.1016/j.yfrne.2022.101013, PMID: 35792198

[22] Giménez-Palomo A Gomes-da-Costa S Dodd S Pachiarotti I Verdolini N Vieta E. Does metabolic syndrome or its component factors alter the course of bipolar disorder? A systematic review. Neurosci Biobehav Rev. (2022) 132:142–53. doi: 10.1016/j.neubiorev.2021.11.026 34800584

[23] Amare AT Schubert KO Klingler-Hoffmann M Cohen-Woods S Baune BT. The genetic overlap between mood disorders and cardiometabolic diseases: a systematic review of genome wide and candidate gene studies. Transl Psychiatry. (2017) 7:e1007. doi: 10.1038/tp.2016.261, PMID: 28117839 PMC5545727

[24] Solmi M Tiihonen J Lähteenvuo M Tanskanen A Correll CU Taipale H. Antipsychotics use is associated with greater adherence to cardiometabolic medications in patients with schizophrenia: results from a Nationwide, within-subject design study. Schizophr Bull. (2022) 48:166–75. doi: 10.1093/schbul/sbab087 34286338 PMC8781351

[25] Leo RJ Singh J. Migraine headache and bipolar disorder comorbidity: a systematic review of the literature and clinical implications. Scand J Pain. (2016) 11:136–45. doi: 10.1016/j.sjpain.2015.12.002, PMID: 28850455

[26] Bschor T Bauer M. Side effects and risk profile of lithium: critical assessment of a systematic review and meta-analysis. Nervenarzt. (2013) 84:860–3. doi: 10.1007/s00115-013-3766-z, PMID: 23525591

[27] Fornaro M Orsolini L Marini S De Berardis D Perna G Valchera A. The prevalence and predictors of bipolar and borderline personality disorders comorbidity: systematic review and meta-analysis. J Affect Disord. (2016) 195:105–18. doi: 10.1016/j.jad.2016.01.040, PMID: 26881339

[28] Frías Á Baltasar I Birmaher B. Comorbidity between bipolar disorder and borderline personality disorder: prevalence, explanatory theories, and clinical impact. J Affect Disord. (2016) 202:210–9. doi: 10.1016/j.jad.2016.05.048, PMID: 27267293

[29] Pavlova B Perlis RH Alda M Uher R. Lifetime prevalence of anxiety disorders in people with bipolar disorder: a systematic review and meta-analysis. Lancet Psychiatry. (2015) 2:710–7. doi: 10.1016/S2215-0366(15)00112-1 26249302

[30] Kauer-Sant'Anna M Frey BN Andreazza AC Ceresér KM Gazalle FK Tramontina J. Anxiety comorbidity and quality of life in bipolar disorder patients. Can J Psychiatr. (2007) 52:175–81. doi: 10.1177/070674370705200309 17479526

[31] Spoorthy MS Chakrabarti S Grover S. Comorbidity of bipolar and anxiety disorders: an overview of trends in research. World J Psychiatry. (2019) 9:7–29. doi: 10.5498/wjp.v9.i1.7, PMID: 30631749 PMC6323556

[32] Watson S Gallagher P Ritchie JC Ferrier IN Young AH. Hypothalamic-pituitary-adrenal axis function in patients with bipolar disorder. Br J Psychiatry. (2004) 184:496–502. doi: 10.1192/bjp.184.6.496 15172943

[33] Bauer M Whybrow PC. Role of thyroid hormone therapy in depressive disorders. J Endocrinol Investig. (2021) 44:2341–7. doi: 10.1007/s40618-021-01600-w, PMID: 34129186 PMC8502157

[34] Giménez-Palomo A Dodd S Anmella G Carvalho AF Scaini G Quevedo J. The role of mitochondria in mood disorders: from physiology to pathophysiology and to treatment. Front Psychol. (2021) 12:546801. doi: 10.3389/fpsyt.2021.546801 PMC8291901 34295268

[35] Kim HK Chen W Andreazza AC. The potential role of the NLRP3 Inflammasome as a link between mitochondrial complex I dysfunction and inflammation in bipolar disorder. Neural Plast. (2015) 2015:408136:1–10. doi: 10.1155/2015/408136 26075098 PMC4444590

[36] Rosenblat JD Brietzke E Mansur RB Maruschak NA Lee Y McIntyre RS. Inflammation as a neurobiological substrate of cognitive impairment in bipolar disorder: evidence, pathophysiology and treatment implications. J Affect Disord. (2015) 188:149–59. doi: 10.1016/j.jad.2015.08.058, PMID: 26363613

[37] Horrobin DF Bennett CN. Depression and bipolar disorder: relationships to impaired fatty acid and phospholipid metabolism and to diabetes, cardiovascular disease, immunological abnormalities, cancer, ageing and osteoporosis. Possible candidate genes. Prostaglandins Leukot Essent Fat Acids. (1999) 60:217–34. doi: 10.1054/plef.1999.0037, PMID: 10397403

[38] Salvadore G Viale CI Luckenbaugh DA Zanatto VC Portela LV Souza DO. Increased uric acid levels in drug-naïve subjects with bipolar disorder during a first manic episode. Prog Neuro-Psychopharmacol Biol Psychiatry. (2010) 34:819–21. doi: 10.1016/j.pnpbp.2010.02.027, PMID: 20206224 PMC3008668

[39] Lachman HM Pedrosa E Petruolo OA Cockerham M Papolos A Novak T. Increase in GSK3beta gene copy number variation in bipolar disorder. Am J Med Genet B Neuropsychiatr Genet. (2007) 144B:259–65. doi: 10.1002/ajmg.b.30498 17357145

[40] Fitzgerald PJ. Noradrenaline transmission reducing drugs may protect against a broad range of diseases. Auton Autacoid Pharmacol. (2015) 34:15–26. doi: 10.1111/aap.12019 25271382

[41] Hajek T Slaney C Garnham J Ruzickova M Passmore M Alda M. Clinical correlates of current level of functioning in primary care-treated bipolar patients. Bipolar Disord. (2005) 7:286–91. doi: 10.1111/j.1399-5618.2005.00182.x, PMID: 15898967

[42] ICD10data.com. (2023). ICD-10-CM codes 2023 Available at: https://www.icd10data.com/ICD10CM/Codes [Accessed March 12, 2023].

[43] Kilbourne AM Cornelius JR Han X Pincus HA Shad M Salloum I. Burden of general medical conditions among individuals with bipolar disorder. Bipolar Disord. (2004) 6:368–73. doi: 10.1111/j.1399-5618.2004.00138.x 15383128

[44] Soreca I Fagiolini A Frank E Houck PR Thompson WK Kupfer DJ. Relationship of general medical burden, duration of illness and age in patients with bipolar I disorder. J Psychiatr Res. (2008) 42:956–61. doi: 10.1016/j.jpsychires.2007.10.009, PMID: 18076906

[45] Rihmer Z Gonda X Döme P. Is mania the hypertension of the mood? Discussion of a hypothesis. Curr Neuropharmacol. (2017) 15:424–33. doi: 10.2174/1570159X14666160902145635, PMID: 28503115 PMC5405605

[46] Chen G Lv H Zhang X Gao Y Liu X Gu C. Assessment of the relationships between genetic determinants of thyroid functions and bipolar disorder: a mendelian randomization study. J Affect Disord. (2022) 298:373–80. doi: 10.1016/j.jad.2021.10.101, PMID: 34728293

[47] Yan X Xu P Sun X. Circadian rhythm disruptions: a possible link of bipolar disorder and endocrine comorbidities. Front Psychol. (2022) 13:1065754. doi: 10.3389/fpsyt.2022.1065754 PMC9849950 36683994

[48] Bartoli F Callovini T Cavaleri D Cioni RM Bachi B Calabrese A. Clinical correlates of comorbid attention deficit hyperactivity disorder in adults suffering from bipolar disorder: a meta-analysis. Aust N Z J Psychiatry. (2023) 57:34–48. doi: 10.1177/00048674221106669, PMID: 35786010

[49] Agnew-Blais J Danese A. Childhood maltreatment and unfavourable clinical outcomes in bipolar disorder: a systematic review and meta-analysis. Lancet Psychiatry. (2016) 3:342–9. doi: 10.1016/S2215-0366(15)00544-1, PMID: 26873185

[50] Joslyn C Hawes DJ Hunt C Mitchell PB. Is age of onset associated with severity, prognosis, and clinical features in bipolar disorder? A meta-analytic review. Bipolar Disord. (2016) 18:389–403. doi: 10.1111/bdi.12419, PMID: 27530107

[51] McDermid J Sareen J El-Gabalawy R Pagura J Spiwak R Enns MW. Co-morbidity of bipolar disorder and borderline personality disorder: findings from the National Epidemiologic Survey on alcohol and related conditions. Compr Psychiatry. (2015) 58:18–28. doi: 10.1016/j.comppsych.2015.01.004, PMID: 25666748

[52] Morris G Puri BK Walker AJ Maes M Carvalho AF Bortolasci CC. Shared pathways for neuroprogression and somatoprogression in neuropsychiatric disorders. Neurosci Biobehav Rev. (2019) 107:862–82. doi: 10.1016/j.neubiorev.2019.09.025, PMID: 31545987

[53] De Hert M Detraux J Vancampfort D. The intriguing relationship between coronary heart disease and mental disorders. Dialogues Clin Neurosci. (2018) 20:31–40. doi: 10.31887/DCNS.2018.20.1/mdehert, PMID: 29946209 PMC6016051

[54] Swardfager W Hennebelle M Yu D Hammock BD Levitt AJ Hashimoto K. Metabolic/inflammatory/vascular comorbidity in psychiatric disorders; soluble epoxide hydrolase (sEH) as a possible new target. Neurosci Biobehav Rev. (2018) 87:56–66. doi: 10.1016/j.neubiorev.2018.01.010 29407524 PMC5860823

